# Directed evolution of the bacteriophage endolysin PlyC reveals a charge-mediated mechanism of thermal stabilization

**DOI:** 10.1016/j.jbc.2026.113393

**Published:** 2026-08-05

**Authors:** Ryan D. Heselpoth, Daniel C. Nelson

**Affiliations:** 1 Institute for Bioscience and Biotechnology Research, University of Maryland, Rockville, Maryland, USA; 2 Laboratory of Bacterial Pathogenesis and Immunology, The Rockefeller University, New York, New York, USA; 3 Department of Veterinary Medicine, University of Maryland, College Park, Maryland, USA

**Keywords:** biophysical characterization, directed evolution, endolysin, kinetic stability, protein engineering, thermal stability

## Abstract

Bacteriophage endolysins are peptidoglycan hydrolases currently being clinically developed as antibacterial therapeutics. Despite possessing several advantageous antibacterial properties, many endolysins display only modest thermal stability. This characteristic hinders their therapeutic potential due to limited long-term stability and complex storage requirements. To overcome this limitation, an evolutionary-based protein engineering strategy was employed in a proof-of-concept study to increase the intrinsic stability of an endolysin. Using the multimeric endolysin PlyC as a model, directed evolution was applied to the thermolabile PlyCA catalytic subunit. After screening 18,000 mutants, the lead candidate identified was the point mutant PlyC (PlyCA_N211H_). The protonated PlyCA_H211_ side-chain stabilizes the subunit by forming favorable electrostatic field interactions with two acidic residues located in the N-terminal glycosyl hydrolase domain, possibly resulting in an extended linker structure being anchored to the surface of the domain. This mutation improved structural and thermal stability under conditions that maximize the stabilizing effect (pH 6.0) by 3.48 °C and 4.10 °C, respectively, and increased kinetic stability 18.6-fold over WT. Combining PlyCA_N211H_ with a stabilizing mutation (PlyCA_T406R_) identified in an independent rational-based *in silico* screen of PlyC additively enhanced thermal stability by 7.46 °C at pH 6.0. Accordingly, with a thermal transition temperature of 54.60 °C, PlyC(PlyCA_N211H,T406R_) now represents a highly stable endolysin derived from a mesophilic phage. In addition to *in silico* screening, this validated directed evolution methodology can now be expanded to other endolysins and bacteriolytic enzymes for the purpose of increasing their thermal stability, thereby improving their practical utility as an antimicrobial biologic.

Double-stranded DNA bacteriophage (phage) utilize a two-component lytic system to liberate progeny virions (1). Late in the phage replication cycle, holins oligomerize in the cytoplasmic membrane of the host bacterium to induce generalized membrane disruption (2, 3). This event allows cytosolic endolysins, which function as peptidoglycan (*i.e.*, cell wall) hydrolases, to bypass the membrane in order to access their peptidoglycan substrates. These enzymes cleave one or more essential covalent bonds in the peptidoglycan to disrupt structural integrity and promote rapid hypotonic lysis, resulting in progeny phage release. When added externally, recombinant endolysins display potent bactericidal activity towards Gram-positive bacteria, thereby highlighting their therapeutic potential as alternatives to small molecule antibiotics (4, 5, 6, 7, 8). Although the physical barrier of the outer membrane of Gram-negative bacteria generally prevents recombinant endolysins from accessing the peptidoglycan when extrinsically applied, there are increasing examples of native and engineered endolysins that are capable of either translocating across (*e.g.*, lysocins) or permeabilizing the outer membrane (*e.g.*, Artilysins) (9, 10, 11, 12, 13, 14, 15, 16, 17). In these instances, the endolysin can access and cleave the peptidoglycan to consequently kill the bacteria.

The streptococcal C_1_ phage endolysin, PlyC, has numerous bacteriolytic properties of clinical interest. Notably, PlyC is the most potent endolysin characterized to date, requiring only nanogram quantities to sterilize a turbid culture of *Streptococcus pyogenes* (group A *Streptococcus*) within seconds (18). In addition to *S. pyogenes*, PlyC can kill species of *Streptococcus* that cause disease in animals, including group C and group E streptococci. Other experimental highlights include potent antibacterial activity against planktonic, biofilm-state, and intracellular streptococci, as well as established *in vivo* antibacterial efficacy using a murine pharyngeal decolonization model (18, 19, 20).

Endolysins that target Gram-positive bacteria are generally 25 to 40 kDa and consist of a single polypeptide with a conserved N-terminal catalytic domain (responsible for peptidoglycan hydrolysis) linked to a variable C-terminal cell wall binding domain (CBD; which dictates bacterial specificity by discriminately binding to a cell wall-associated epitope) (6). Conversely, PlyC possesses a unique multimeric structure consisting of nine subunits (Fig. 1) (21, 22). The catalytic subunit, PlyCA, consists of an N-terminal glycosyl hydrolase (GyH) domain (Fig. 1*A*, blue), a central helical docking domain (Fig. 1*A*, yellow), and a C-terminal cysteine, histidine-dependent amidohydrolase/peptidase (CHAP) domain (Fig. 1*A*, red). The GyH domain functions synergistically with the CHAP domain to generate robust bacteriolytic activity, while the helical docking domain interacts with the PlyCB CBD (Fig. 1*A*, gray) to form a structurally complex but highly potent holoenzyme (21). The PlyCB CBD comprises eight PlyCB monomers that oligomerize to form an octameric ring structure that binds to polyrhamnose epitopes shared in the wall teichoic acids of sensitive species of *Streptococcus* (Fig. 1*B*) (23, 24).

**Figure 1:**
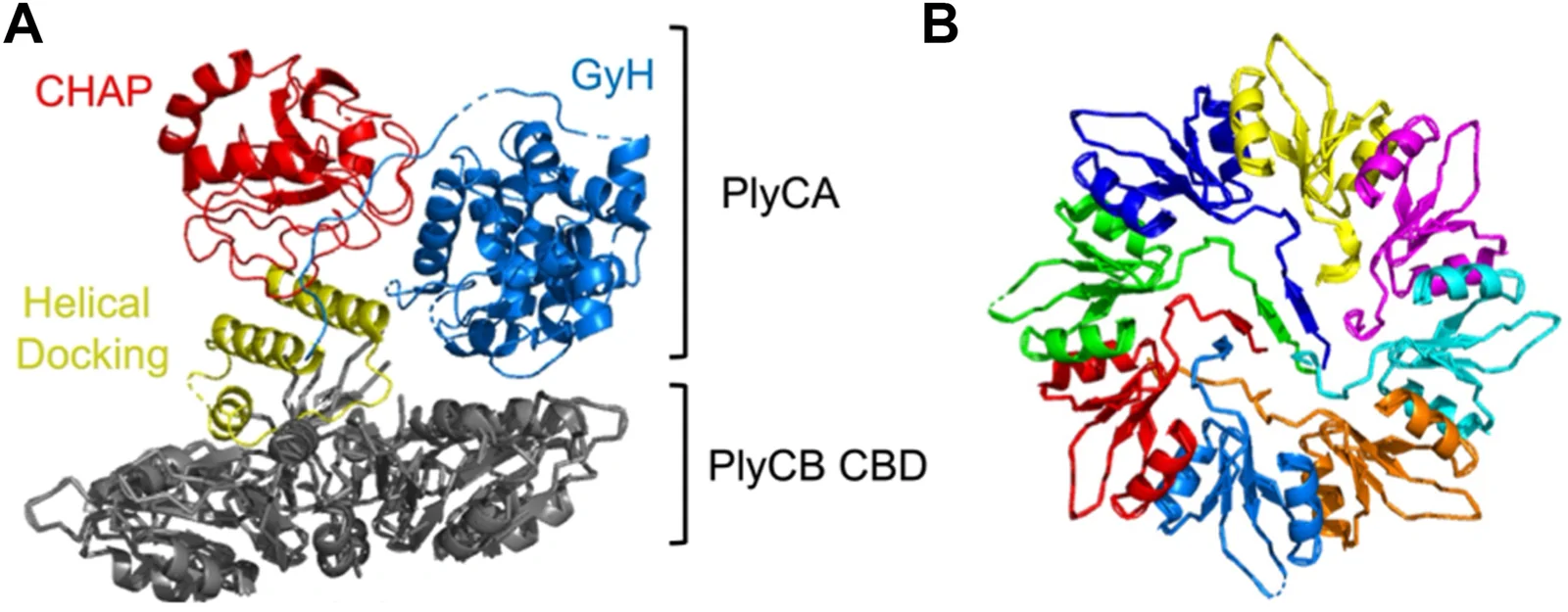
**Structural organization of the PlyC holoenzyme.***A,* PlyC is composed of nine subunits, including a single catalytic PlyCA subunit and an octameric PlyCB CBD. PlyCA comprises an N-terminal GyH domain (*blue*), a central helical docking domain (*yellow*) and a CHAP domain (*red*). The helical docking domain mediates interactions between PlyCA and the PlyCB CBD (*gray*) to form the holoenzyme. *B,* the PlyCB CBD consists of eight identical PlyCB subunits (shown in different colors) that assemble into an octameric ring structure. CHAP, cysteine, histidine-dependent amidohydrolase/peptidase; GyH, glycosyl hydrolase; CBD, cell wall binding domain.

Despite its attractive antibacterial properties, the practical development of PlyC as a protein therapeutic is hindered by its modest intrinsic thermal stability (25, 26, 27). Differential scanning calorimetry (DSC) confirmed that the PlyCA subunit is thermally denatured between 46 °C and 48 °C. This denaturation event causes the irreversible kinetic inactivation of the endolysin. In contrast to PlyCA, the PlyCB CBD is endogenously thermostable, with the structural integrity of the CBD remaining intact until heated to 75 °C. Although additional studies are necessary, data obtained from an initial thermal evaluation of the PlyC holoenzyme and various PlyC-derived structural components (PlyCA, the C-terminal CHAP domain of PlyCA, and the PlyCB CBD) indicate that the CHAP domain could be the least thermostable segment of the endolysin (27). The inherent thermolability of PlyC is comparable to that of other endolysins originating from mesophilic phage. For example, the *Staphylococcus aureus* endolysins LysK and PlyGRCS, as well as the *Streptococcus pneumoniae* endolysins Cpl-1 and ClyJ, are kinetically inactivated and/or unfold at temperatures below 44 °C (28, 29, 30, 31). Additionally, the *S. aureus* endolysins LysPhi80α and ClyC experience a ∼50% reduction in residual lytic activity when incubated at 37 °C and 45 °C, respectively (32, 33). Considering the thermal properties of protein therapeutics are highly relevant to their practical development, the intrinsic instability of various endolysins, like PlyC, could impede the downstream therapeutic potential of the biologic (34).

Numerous protein engineering methodologies can potentially be implemented for the purpose of designing modified endolysins with increased thermal stability. One such engineering approach is directed evolution, which uses the concept of Darwinian evolution in a laboratory setting to alter various macromolecular properties (35, 36). This methodology involves first introducing molecular diversity to a target protein or enzyme using random mutagenesis and *in vitro* recombination, followed by a stringent screening process to identify variants with progressed fitness. After several iterations of mutagenesis, screening and amplification of selected mutants with improved properties, beneficial mutations accumulate in true Darwinian evolution (37). Directed evolution is commonly practiced for increasing protein stability and is often preferred to other engineering strategies due to the level of diversification and size of the mutant pool screened. Moreover, neither structural nor mechanistic information are required to design and implement these experiments, making directed evolution particularly well suited for complex enzymes where rational stabilization strategies are not immediately apparent. There are examples of directed evolution being used to enhance enzyme thermal stability. For example, this approach improved the kinetic stability of subtilisin and a β-glucosidase 1200- and 8640-fold, respectively, and increased the melting temperatures of a cellulase, D-allulose 3-epimerase, esterase, and cold-shock protein by a respective 10.4 °C, 14 °C, 14 °C, and 28 °C (38, 39, 40, 41, 42, 43).

In this proof-of-concept study, we sought to increase the thermal stability of the model endolysin PlyC using a directed evolution strategy. This approach employed iterative rounds of random mutagenesis using the error-prone polymerase chain reaction (epPCR) followed by selection, a well-established methodology in protein engineering. We initially performed a comprehensive calorimetric evaluation of PlyC and all its structural components to conclusively elucidate the least thermostable region(s) of the endolysin. Once identified, this region was strategically screened for stabilizing mutations using a previously developed and validated directed evolution protocol optimized for increasing the stability of bacteriolytic enzymes (25). The lead candidate at the end of the screening process was thermally characterized to determine mechanistically how this evolved PlyC mutant exhibits improved thermal stability. Finally, a stabilizing point mutation identified in an independent rational-based *in silico* screen of PlyC (26) was integrated into the directed evolution lead candidate to further expand thermal stability in an additive manner, yielding one of the more thermostable endolysins described to date originating from a mesophilic phage.

## Results

### Thermal stability of the structural constituents of PlyC

A previous thermal analysis of PlyC indicated the C-terminal CHAP domain of PlyCA could be the most thermolabile structural component of the holoenzyme (27). To expand on this finding, additional PlyC constructs were calorimetrically characterized. In addition to the previously studied PlyC, PlyCA, CHAP domain of PlyCA, and the PlyCB CBD, other constructs evaluated include the N-terminal GyH domain of PlyCA, PlyCΔGyH (*i.e.,* PlyC holoenzyme with the GyH domain of PlyCA deleted), PlyCΔCHAP (*i.e.,* PlyC holoenzyme with the CHAP domain of PlyCA deleted), and PlyCΔΔ (*i.e.,* PlyC holoenzyme with both the GyH and CHAP domains of PlyCA deleted) (Table S1). Each PlyC construct was expressed as a recombinant protein in *Escherichia coli* and subsequently purified (Fig. S1). Using DSC, the thermal stability of each purified protein was then determined at equal molar concentrations in 20 mM sodium phosphate buffer, pH 7.0. All samples were heated from 15 °C to 105 °C at 1 °C/min, and then immediately cooled over the same temperature range.

Consistent with previous studies, the thermal stability of PlyCA and the PlyCB CBD differ significantly. In the context of the holoenzyme, which fulfilled a five-state thermal transition model, the thermal transition temperature (*T*_*G*_) of PlyCA (*T*_*G*_ = 48.27 °C) is 27.58 °C lower than that of the PlyCB CBD (*T*_*G*_ = 75.85 °C) (Table 1, Fig. S2). When isolated, PlyCA (*T*_*G*_ = 45.92 °C) was destabilized relative to its complexed form, exhibiting a thermal transition temperature 29.52 °C lower than the isolated PlyCB CBD (*T*_*G*_ = 75.44 °C) (Table 1, Figs. S3 and S4). Calorimetric analysis of the isolated GyH (*T*_*G*_ = 47.74 °C) and CHAP domains (*T*_*G*_ = 47.81 °C) of PlyCA revealed both domains displayed near-identical thermal transition temperatures (Table 1, Figs. S5 and S6). When complexed with the PlyCB CBD, deletion of the GyH domain from PlyCA increased the stability of the catalytic subunit by 1.34 °C (*T*_*G*_ = 49.61 °C), whereas removal of the CHAP domain marginally destabilized PlyCA by 0.43 °C (*T*_*G*_ = 47.84 °C) (Table 1, Figs. S7 and S8). A distinct thermal transition temperature for the central helical docking domain was not detected following a calorimetric analysis of the PlyCΔΔ construct (Table 1, Fig. S9). This observation may reflect the concurrent unfolding of the helical docking domain and the PlyCB CBD. After heating each sample, results from the subsequent cooling step showed each sample unfolds irreversibly (data not shown).

**Table 1:** Calorimetric thermal transition temperature analysis of the PlyCA (columns 2 and 3) and PlyCB cell wall binding domain (columns 4 and 5) components of each PlyC construct in 20 mM sodium phosphate buf, pH 7.0

PlyC construct	PlyCA		PlyCB	
	*T*_*G1*_ (°C)	*T*_*G2*_ (°C)	*T*_*G3*_ (°C)	*T*_*G4*_ (°C)
PlyC	48.27	50.67	75.85	79.32
PlyCA	45.92	48.41	n/a	n/a
PlyCB	n/a	n/a	75.44	77.00
Glycosyl hydrolase domain	47.74	49.44	n/a	n/a
Cysteine, histidine-dependent amidohydrolase/peptidase domain	47.81	50.14	n/a	n/a
PlyCΔGlycosyl hydrolase domain	49.61	56.77	73.90	75.73
PlyCΔCysteine, histidine-dependent amidohydrolase/peptidase domain	47.84	50.22	76.26	79.52
PlyCΔΔ	n/d	n/d	74.23	76.59

### Directed evolution screening synopsis

**Figure 2:**
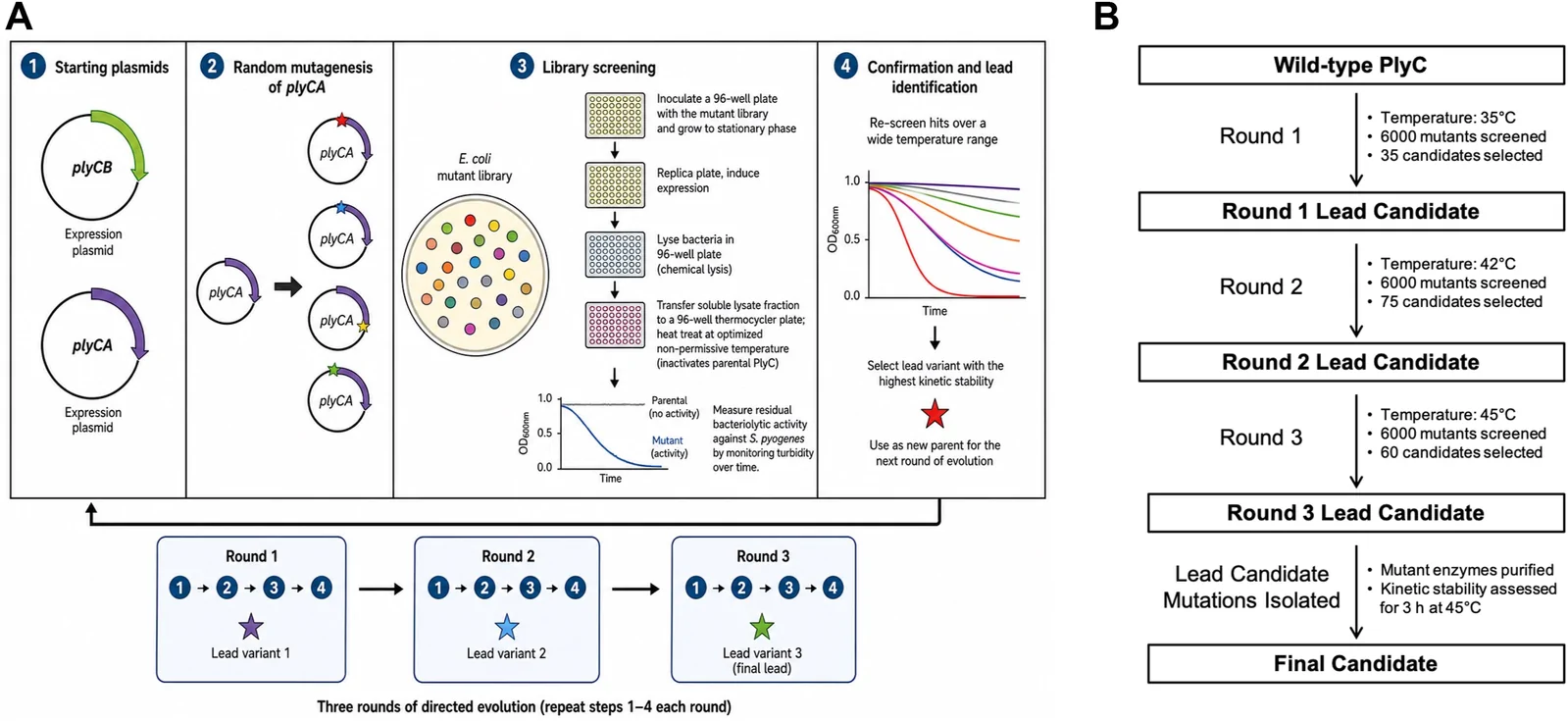
**Overview of the PlyC directed evolution methodology.***A,* mutant *plyCA* libraries generated by error-prone polymerase chain reaction were coexpressed with PlyCB in *E. coli*, heat challenged under nonpermissive conditions and screened for residual bacteriolytic activity against *S.pyogenes*. Lead variants exhibiting improved kinetic stability were used as a template for subsequent rounds of evolution. *B,* a total of 18,000 PlyC mutants was screened over three rounds of directed evolution. The *E. coli* soluble lysates containing the overexpressed PlyC mutants were treated with the following optimized temperatures: Round 1: 35 °C, Round 2: 42 °C, and Round 3: 45 °C. Mutants with confirmed enhanced stability were then rescreened over a wide temperature range, with the variant displaying residual activity at the highest temperature being labeled as the lead candidate for the next round of screening. After the third round of directed evolution, the four amino acid mutations encoded by the evolved lead candidate were isolated by site-directed mutagenesis. The most kinetically stable PlyC mutant was labeled as the overall lead candidate.

Because the uncomplexed and complexed GyH and CHAP domains exhibited comparable thermal stability profiles, and calorimetric analysis of the central helical docking domain was inconclusive, the entire PlyCA subunit was targeted for directed evolution. A schematic of the screening workflow is shown in Figure 2*A*. Briefly, epPCR was used to generate mutant *plyCA* libraries, which were coexpressed with PlyCB in *E. coli* to produce functional PlyC holoenzyme variants. Following protein expression, soluble lysates containing the mutant enzymes were subjected to heat treatment at a non-permissive temperature optimized to inactivate the parental PlyC enzyme. Residual bacteriolytic activity against *S. pyogenes* was then measured to identify variants with improved kinetic stability. Putative hits were re-screened using a range of heat-treatment temperatures to eliminate false positives and identify the lead variant with the most progressed thermal tolerance. The lead variant from each round was then used as the template for the subsequent round of epPCR.

Overall, 18,000 PlyC mutants were screened for enhanced kinetic stability over a total of three rounds of directed evolution (Fig. 2*B*). For round one, the template for epPCR was WT *plyCA*. After screening 6,000 PlyC mutants at 35 °C, 35 candidates were selected, re-screened to remove false positives, and then assayed for residual bacteriolytic activity over a broad temperature range to identify a lead candidate. For round two of directed evolution, epPCR was applied to the *plyCA* template encoding the lead candidate from round one. Following the screening of an additional 6,000 second-generation PlyC mutants at 42°C, a total of 75 mutant candidates were chosen and re-screened. Once a lead candidate was selected, the *plyCA* template encoding this particular mutant was targeted by epPCR for the final round of directed evolution. The final 6,000 third-generation PlyC mutants were screened at 45 °C, with 60 candidates being identified for re-screening. The lead candidate at the conclusion of the third round of directed evolution was termed PlyC 33D2, which collectively encoded the four PlyCA amino acid mutations N211H, S224N, G249C and A407S.

### Kinetic stability of PlyC 33D2 and PlyC 33D2-derived point mutants

The thermally-induced kinetic half-life (*t*_*1/2*_) of PlyC 33D2 was determined and compared to that of WT in phosphate buffered saline (PBS), pH 7.2. For these and subsequent experiments evaluating kinetic stability, each purified endolysin (Fig. S10) was incubated at equal concentrations for a total of 3 h at 45°C. Aliquots were removed in 20 min increments, cooled on ice and assayed for residual lytic activity against *S. pyogenes*. Data were normalized to the lytic activity of an unheated control. The kinetic half-life, *t*_*1/2*_, corresponds to the amount of time (min) required to reduce the relative bacteriolytic activity of the endolysin to 50%.

**Figure 3:**
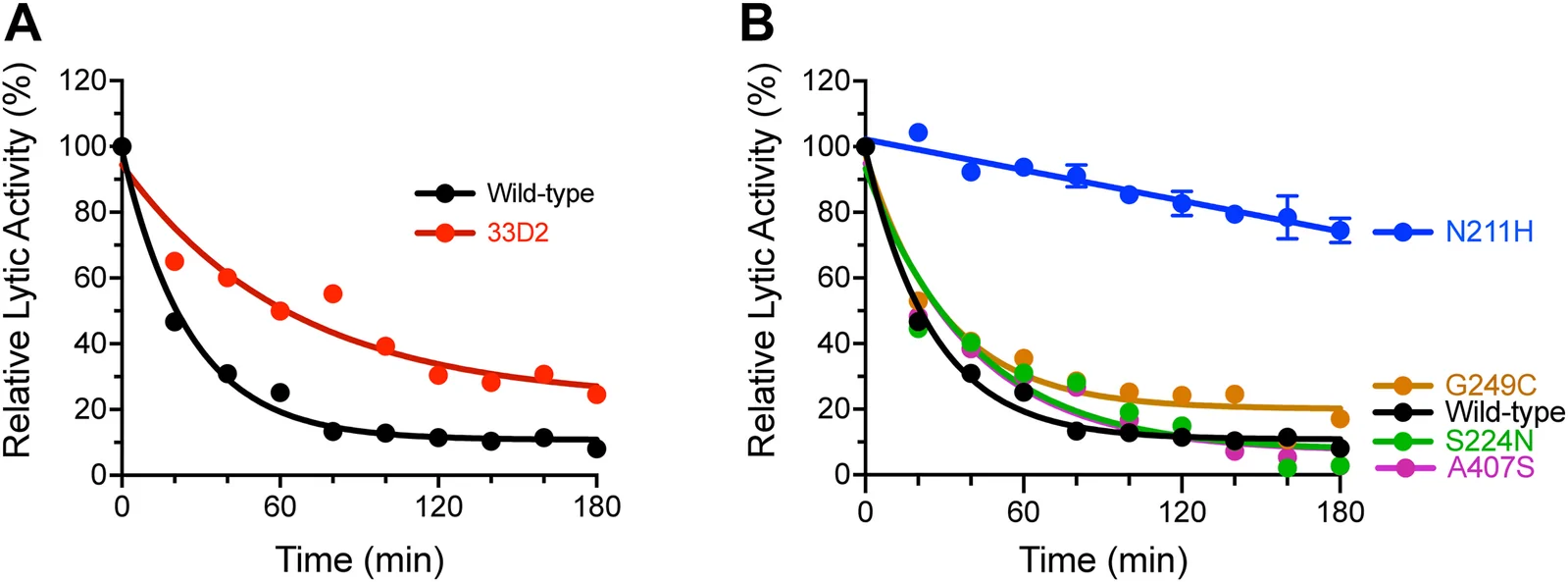
**Kinetic stability of PlyC 33D2 and PlyC 33D2-derived point mutants.** The kinetic stability of WT PlyC was compared to that of either *(A)* the round 3 lead candidate, PlyC 33D2, or *(B)* PlyC 33D2-derived point mutants. The enzymes at equal concentrations were incubated at 45 °C in PBS; pH 7.2, with the residual bacteriolytic activity assayed against *S. pyogenes* every 20 min for a total of 3 h. All data were normalized to the activity displayed by the unheated samples. Error bars correspond to the ± standard error of the mean of triplicate experiments. All protein samples analyzed were purified to homogeneity (Fig. S10). PBS, phosphate buffered saline.

At 45 °C, the PlyC 33D2 mutant displayed a *t*_*1/2*_ of 45 min, representing a 2.5-fold increase in kinetic stability when compared to WT PlyC (*t*_*1/2*_ = 18 min) (Fig. 3*A*, Table 2). Next, each mutation collectively encoded by PlyC 33D2 was isolated in order to evaluate the effect each had on the kinetic stability of the endolysin. PlyC (PlyCA_S224N_), PlyC (PlyCA_G249C_), and PlyC (PlyCA_A407S_) had similar *t*_*1/2*_ values at 45°C of 28, 21, and 27 min, respectively, representing between a 1.2- to 1.6-fold increase in kinetic stability over WT (Fig. 3*B*, Table 2). In contrast, the *t*_*1/2*_ of PlyC (PlyCA_N211H_) was an extrapolated 335 min, correlating to an 18.6-fold improvement in kinetic stability when compared to WT. The kinetic stability of PlyC(PlyCA_N211H_) was 7.4-fold higher than that of PlyC 33D2, suggesting that combining PlyCA_N211H_ with one or more of the additional PlyC 33D2 mutations reduces kinetic stability.

**Table 2:** Kinetic half-life comparison between WT PlyC, PlyC 33D2, and PlyC 33D2 point mutants at 45 °C in phosphate buffered saline, pH 7.2

PlyC construct	*t*_*1/2*_ (min)	Relative kinetic stability	R^2^
WT	18	1.0	0.99
33D2	45	2.5	0.93
N211H	335	18.6	0.94
S224N	28	1.6	0.93
G249C	21	1.2	0.96
A407S	27	1.5	0.96

### Thermal stability of the directed evolution lead candidate

**Figure 4:**
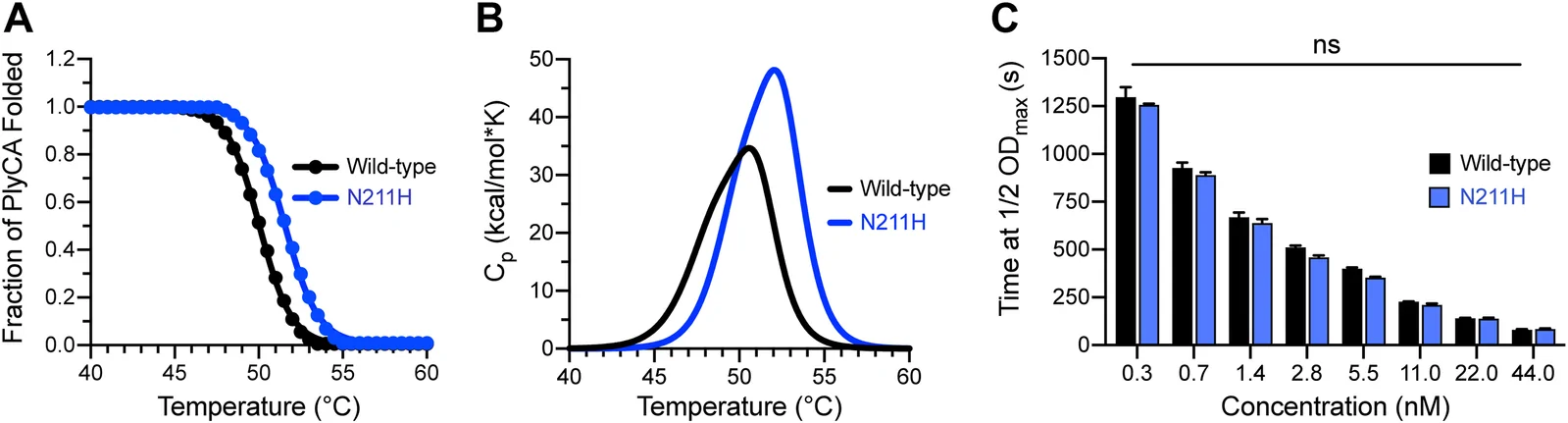
**Thermal analysis and bacteriolytic activity of PlyC (PlyCA_N211H_).** Biophysical studies involving *(A)* CD thermal denaturation experiments and *(B)* DSC were used to determine the thermal stability of WT PlyC and PlyC (PlyCA_N211H_) in 20 mM sodium phosphate buffer, pH 7.0, using a heating rate of 1 °C/min. The structural and thermal transitions depicted correspond to the unfolding of the PlyCA subunit only. *C,* at equal molar concentrations, the bacteriolytic activity of PlyC (PlyCA_N211H_) was compared to that of WT PlyC in PBS using a dose-response turbidity reduction assay. Activity was quantitated as the amount of time (s) required for each endolysin sample to decrease the initial optical density of the *S. pyogenes* culture by 50%. Error bars represent the ± standard error of the mean of triplicate experiments. *p-*values were calculated using a two-way ANOVA Sidak multiple comparison test. ns, not significant. CD, circular dichroism; DSC, differential scanning calorimetry; PBS, phosphate buffered saline.

The thermal stability of both WT PlyC and PlyC (PlyCA_N211H_) were investigated in 20 mM sodium phosphate buffer, pH 7.0. CD thermal denaturation experiments using PlyC result in PlyCA exhibiting a single cooperative thermally-induced structural transition. The temperature at which the concentration of the folded and unfolded states of PlyCA was equal was defined as the structural midpoint temperature (*T*_*1/2*_). For WT PlyC and PlyC (PlyCA_N211H_), the respective *T*_*1/2*_ values for PlyCA were 50.09 °C and 51.61 °C (Fig. 4*A*, Table 3). The PlyCA_N211H_ mutation therefore improved the structural stability of the catalytic subunit by 1.52 °C at pH 7.0.

**Table 3:** Thermal stability of the different PlyC (PlyCA_N211_) charge mutants using CD thermal denaturation experiments (columns 2 and 3) and differential scanning calorimetry (columns 4–7) in 20 mM sodium phosphate buffer, pH 7.0

PlyC construct	Circular dichroism		Differential scanning calorimetry			
	*T*_*1/2*_ (°C)	*ΔT*_*1/2*_ (°C)	*T*_*G1*_ (°C)	*T*_*G2*_ (°C)	Δ*H*_vH1_ (kcal/mol)	Δ*H*_vH2_ (kcal/mol)
WT	50.09	-	48.27	50.67	171.23	205.09
N211H	51.61	+1.52	50.26	52.46	199.25	240.14
N211A	50.10	+0.01	47.78	50.45	186.38	205.45
N211K	51.21	+1.12	49.61	52.16	170.97	223.74
N211D	46.10	−3.99	45.19	48.63	117.22	161.61

Calorimetric analysis of WT PlyC and PlyC (PlyCA_N211H_) confirmed the PlyCA_N211H_ mutation (*T*_*G*_ = 50.26 °C, van't Hoff enthalpy of unfolding (Δ*H*_vH_) = 199.25 kcal/mol) increases the thermal stability of the catalytic subunit 1.99 °C when compared to WT (*T*_*G*_ = 48.27 °C, Δ*H*_vH_ = 171.23 kcal/mol) (Fig. 4*B*, Table 3). Although unfolding of the catalytic subunit was irreversible, previous DSC analysis demonstrated that the transition temperature and unfolding enthalpy of PlyCA were scan-rate independent, suggesting minimal kinetic distortion under the conditions tested (27). Accordingly, the van't Hoff enthalpies are reported as apparent values. The collective results from these biophysical experiments demonstrate PlyC (PlyCA_N211H_) has improved thermal stability relative to WT.

Using a dose-response turbidity reduction assay, the bacteriolytic activity of WT PlyC was compared to that of PlyC (PlyCA_N211H_) to assess if the PlyCA_N211H_ mutation had any effect on activity. Results from these experiments indicate that the activities of the two enzymes are highly comparable across all molar concentrations assayed (Fig. 4*C*). Based on these findings, PlyC (PlyCA_N211H_) was designated as the lead candidate moving forward.

### PlyCA_H211_ employs a charge-mediated stabilizing mechanism

**Figure 5:**
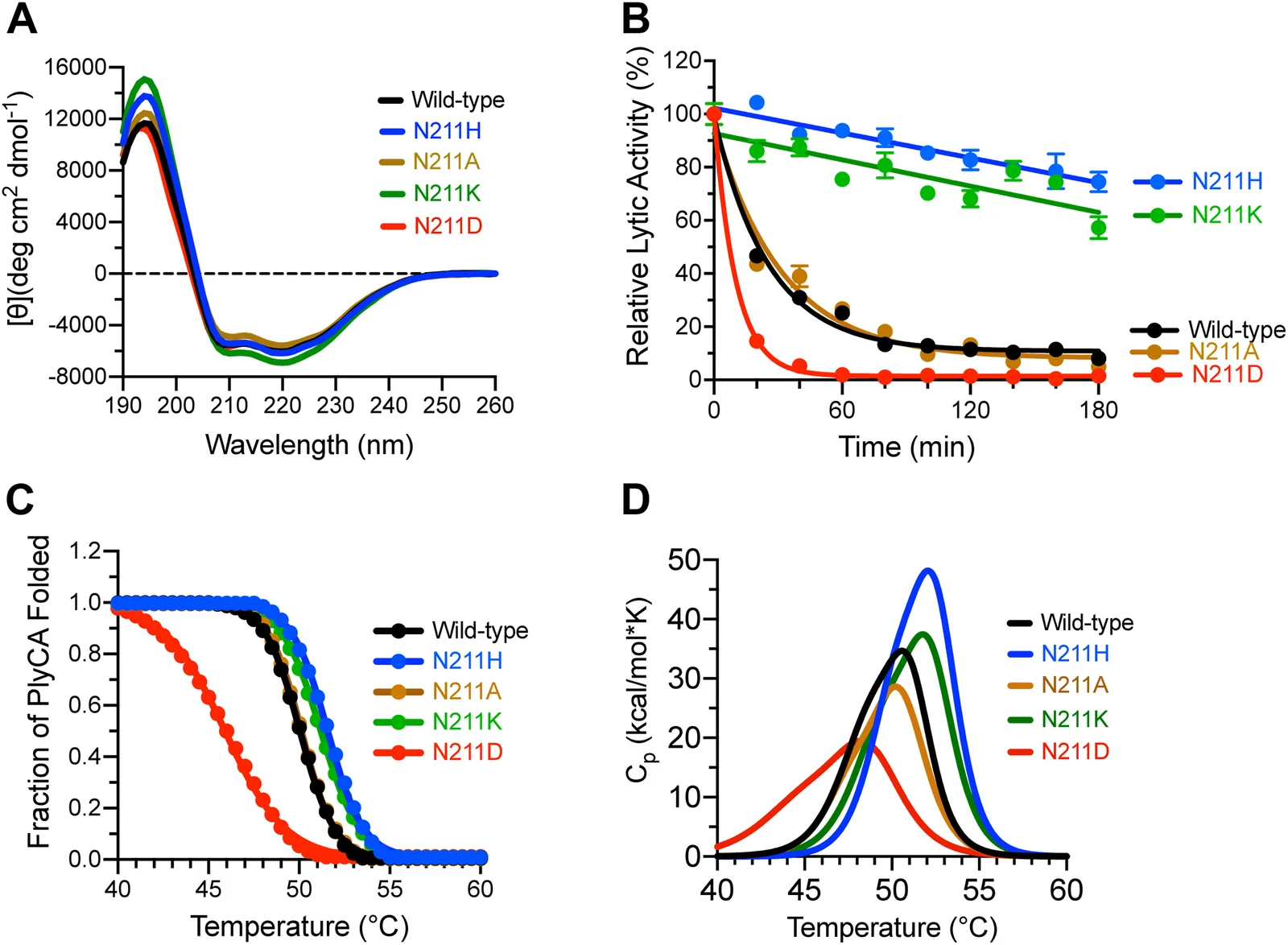
**Thermal stability of charge-specific PlyC (PlyCA_N211_) mutants.***A,* far-UV CD spectroscopy was used to assess the secondary structure composition of WT PlyC and the various PlyC (PlyCA_N211_) charge mutants in 20 mM sodium phosphate buffer, pH 7.0. *B,* the kinetic stability of PlyC (PlyCA_N211A_), PlyC (PlyCA_N211K_), and PlyC (PlyCA_N211D_) was measured over 3 h at 45 °C. Each endolysin sample was incubated in PBS, pH 7.2, with the residual bacteriolytic activity assayed against *S. pyogenes* every 20 min. All data were normalized to the lytic activity displayed by the unheated samples. Error bars correspond to the ± standard error of the mean of triplicate experiments. *C,* CD thermal denaturation and *(D)* DSC were used to determine the thermal stability of PlyC (PlyCA_N211A_), PlyC(PlyCA_N211K_), and PlyC(PlyCA_N211D_). Both experiments were performed in 20 mM sodium phosphate buffer, pH 7.0, using a heating rate of 1 °C/min. The structural and thermal transitions shown pertain to the unfolding of the PlyCA subunit only. All protein samples analyzed were purified to homogeneity (Fig. S10). For *B–D*, previously obtained data for WT PlyC and PlyC (PlyCA_N211H_) are shown for reference purposes. CD, circular dichroism; DSC, differential scanning calorimetry; PBS, phosphate buffered saline.

For PlyC (PlyCA_N211H_), the mechanistic basis by which a histidine at residue 211 increases the thermal stability of PlyCA remains unclear. Considering the mutation consisted of substituting an asparagine with a histidine, we hypothesized the possible addition of the protonated histidine side-chain could increase the stability of the catalytic subunit using a charge-mediated mechanism. To test this hypothesis, we performed a charge-dependent mutational thermal analysis focusing on residue 211 of PlyCA. The thermal properties of three mutants, specifically PlyC (PlyCA_N211A_), PlyC (PlyCA_N211K_), and PlyC (PlyCA_N211D_), were elucidated and compared to the WT PlyC and PlyC (PlyCA_N211H_) controls. Far-UV CD spectroscopy confirmed WT PlyC, PlyC(PlyCA_N211H_), PlyC (PlyCA_N211A_), PlyC (PlyCA_N211K_), and PlyC(PlyCA_N211D_) have the same secondary structure composition (Fig. 5*A*). Therefore, without any significant structural alterations, differences in thermal stability can be directly attributed to the charged state of residue 211 of PlyCA.

When analyzing kinetic stability at 45 °C in PBS, the neutral-charged mutant, PlyC (PlyCA_N211A_), displayed a *t*_*1/2*_ of 22 min, which resembles that of WT PlyC (*t*_*1/2*_ = 18 min) (Fig. 5*B*). The positively-charged mutant, PlyC (PlyCA_N211K_), had an extrapolated *t*_*1/2*_ of 259 min, representing a 14.4-fold increase in kinetic stability over WT. In contrast, the negatively-charged mutant, PlyC (PlyCA_N211D_), was kinetically destabilized 2.6-fold compared to WT, exhibiting a *t*_*1/2*_ of 7 min. These results indicate that replacing PlyCA_N211_ with a cationic amino acid, such as PlyCA_N211H_ or PlyCA_N211K_, improves the kinetic stability of the catalytic subunit in a charge-dependent manner.

To further establish the charge-based effect residue 211 has on the stability of PlyCA, we investigated the thermal stability of the charge mutants in 20 mM sodium phosphate buffer, pH 7.0. Results from CD thermal denaturation experiments of PlyC (PlyCA_N211A_), PlyC (PlyCA_N211K_), and PlyC (PlyCA_N211D_) yielded respective *T*_*1/2*_ values for PlyCA of 50.10 °C, 51.21 °C and 46.10 °C, representing a +0.01°C, +1.12 °C and −3.99 °C difference in structural stability when compared to WT (*T*_*1/2*_ = 50.09 °C) (Fig. 5*C*, Table 3). Calorimetric analysis of PlyC (PlyCA_N211A_), PlyC (PlyCA_N211K_), and PlyC (PlyCA_N211D_) resulted in respective PlyCA thermal transition temperatures of 47.78 °C, 49.61 °C and 45.19 °C, equating to a −0.49 °C, +1.34 °C and −3.08 °C change in thermal stability compared to WT (*T*_*G*_ = 48.27 °C) (Fig. 5*D*, Table 3). Altogether, the biophysical experimental results indicate that the increased thermal stability displayed by PlyC (PlyCA_N211H_) is mediated through a charge-dependent mechanism.

### Effect of the protonated state of PlyCA_H211_ on thermal stability

Because replacement of residue 211 with a cationic amino acid improves stability, altering the protonation state of the H211 side-chain in PlyC (PlyCA_N211H_) should modulate its stabilizing effect relative to WT PlyC. To test this hypothesis, the thermal stability of both WT PlyC and PlyC (PlyCA_N211H_) were measured in 20 mM phosphate buffer, pH 6.0 to 8.0. Under these experimental conditions, the histidine side-chain will be in its most protonated state at pH 6.0 and will become increasingly deprotonated as the pH of the buffer transitions towards 8.0.

**Figure 6:**
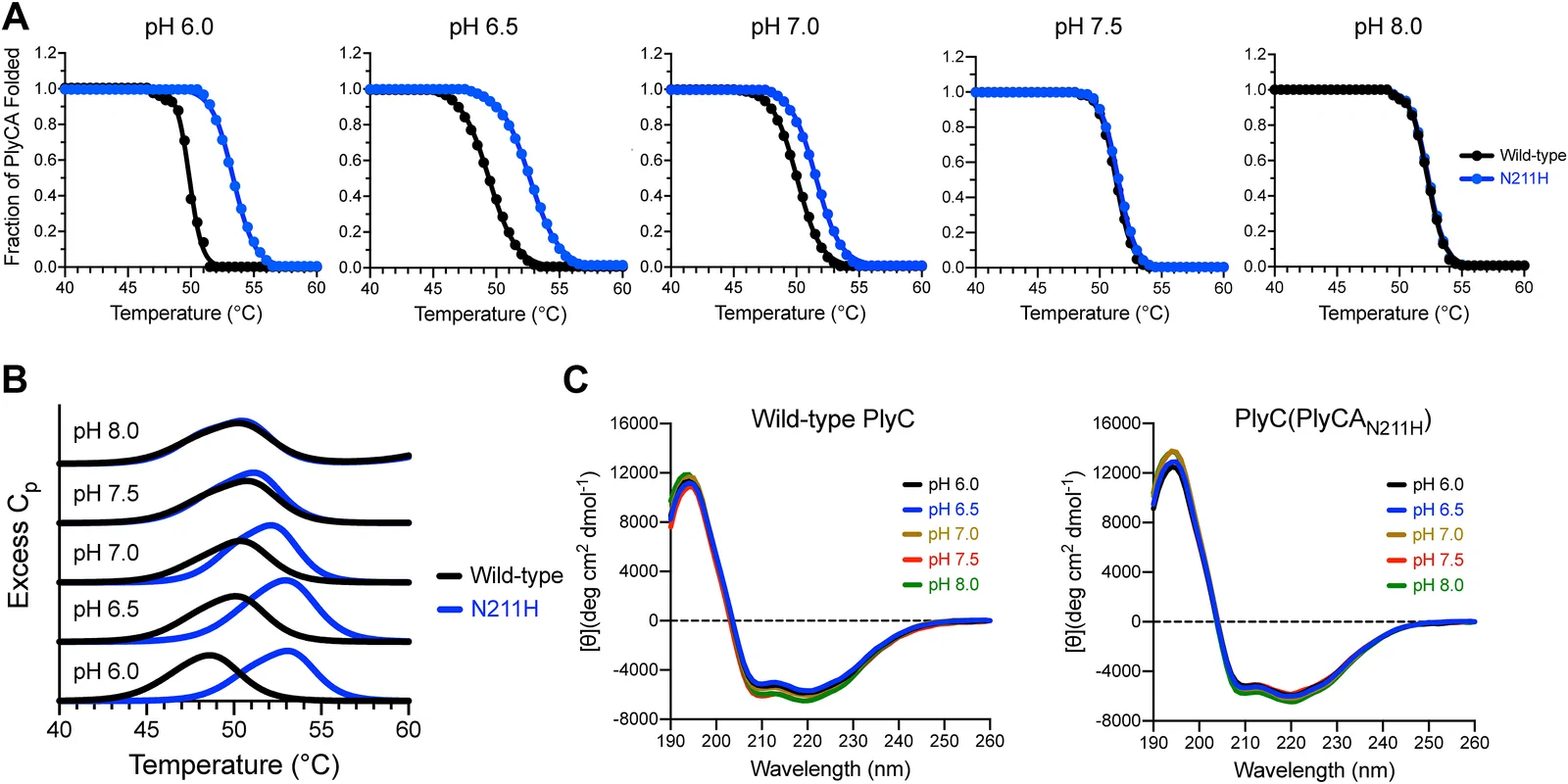
**pH-dependent thermal stability of WT PlyC and PlyC(PlyCA_N211H_).** The effect of pH on the thermal stability of WT PlyC and PlyC (PlyCA_N211H_) was elucidated by heating each sample at 1 °C/min in 20 mM sodium phosphate buffer, pH 6.0 to 8.0. The resulting *(A)* CD thermal denaturation data and *(B)* overlaid DSC thermograms specific to the unfolding of the PlyCA subunit are shown. *C,* the influence of pH on the structure of the endolysin was evaluated by obtaining far-UV CD spectra from 190 to 260 nm for WT PlyC and PlyC (PlyCA_N211H_) in 20 mM sodium phosphate buffer, pH 6.0 to 8.0. CD, circular dichroism; DSC, differential scanning calorimetry.

At pH 6.0, CD thermal denaturation results showed PlyC (PlyCA_N211H_) exhibits a Δ*T*_*1/2*_ for PlyCA of +3.48 °C over WT PlyC (Fig. 6*A*, Table 4). Results from similar experiments at pH 6.5, 7.0, 7.5, and 8.0 revealed corresponding Δ*T*_*1/2*_ values for PlyCA of +3.23 °C, +1.47 °C, +0.21 °C, and +0.08 °C, respectively, compared to WT. The same trend was observed when using DSC. At pH 6.0, PlyC (PlyCA_N211H_) displayed a Δ*T*_*G*_ for PlyCA of +4.10 °C over WT (Fig. 6*B*, Table 5). At pH 6.5, 7.0, 7.5, and 8.0, PlyC (PlyCA_N211H_) respectively had a Δ*T*_*G*_ for PlyCA of +3.20 °C, +1.99 °C, +0.48 °C, and +0.21 °C when compared to WT. Far-UV CD spectroscopy confirmed that varying the buffer pH from 6.0 to 8.0 did not change the secondary structure composition of WT PlyC and PlyC (PlyCA_N211H_), thus eliminating the possibility of significant pH-induced structural changes influencing stability (Fig. 6*C*). Accordingly, as the H211 side-chain becomes increasingly deprotonated to approximate the neutral charge characteristics of N211, the stability of PlyC (PlyCA_N211H_) is progressively comparable to that of WT.

**Table 4:** pH-dependent structural stability of PlyCA in WT PlyC and PlyC (PlyCA_N211H_) backgrounds in 20 mM sodium phosphate buffer, pH 6.0 to 8.0

pH	WT	N211H	*ΔT*_*1/2*_
	*T*_*1/2*_ (°C)	*T*_*1/2*_ (°C)	
6.0	49.88	53.36	+3.48
6.5	49.42	52.65	+3.23
7.0	50.09	51.56	+1.47
7.5	51.30	51.51	+0.21
8.0	52.24	52.32	+0.08

**Table 5:** pH-dependent thermal stability of WT PlyC and PlyC (PlyCA_N211H_) in 20 mM sodium phosphate buffer, pH 6.0 to 8.0

pH	WT				N211H			
	PlyCA		PlyCB		PlyCA		PlyCB	
	*T*_*G1*_ (°C)	*T*_*G2*_ (°C)	*T*_*G3*_ (°C)	*T*_*G4*_ (°C)	*T*_*G1*_ (°C)	*T*_*G2*_ (°C)	*T*_*G3*_ (°C)	*T*_*G4*_ (°C)
6.0	47.14	49.05	82.56	84.26	51.24	53.49	82.94	84.34
6.5	48.28	50.53	80.59	85.01	51.48	53.50	80.70	85.23
7.0	48.27	50.67	75.85	79.32	50.26	52.46	76.18	79.90
7.5	48.64	51.13	70.15	74.51	49.12	51.46	70.66	74.95
8.0	48.14	50.76	66.32	70.49	48.35	50.93	66.44	70.58

### PlyCA_H211_ electrostatically interacts with PlyCA_E113_ and PlyCA_D150_ of the GyH domain

**Figure 7:**
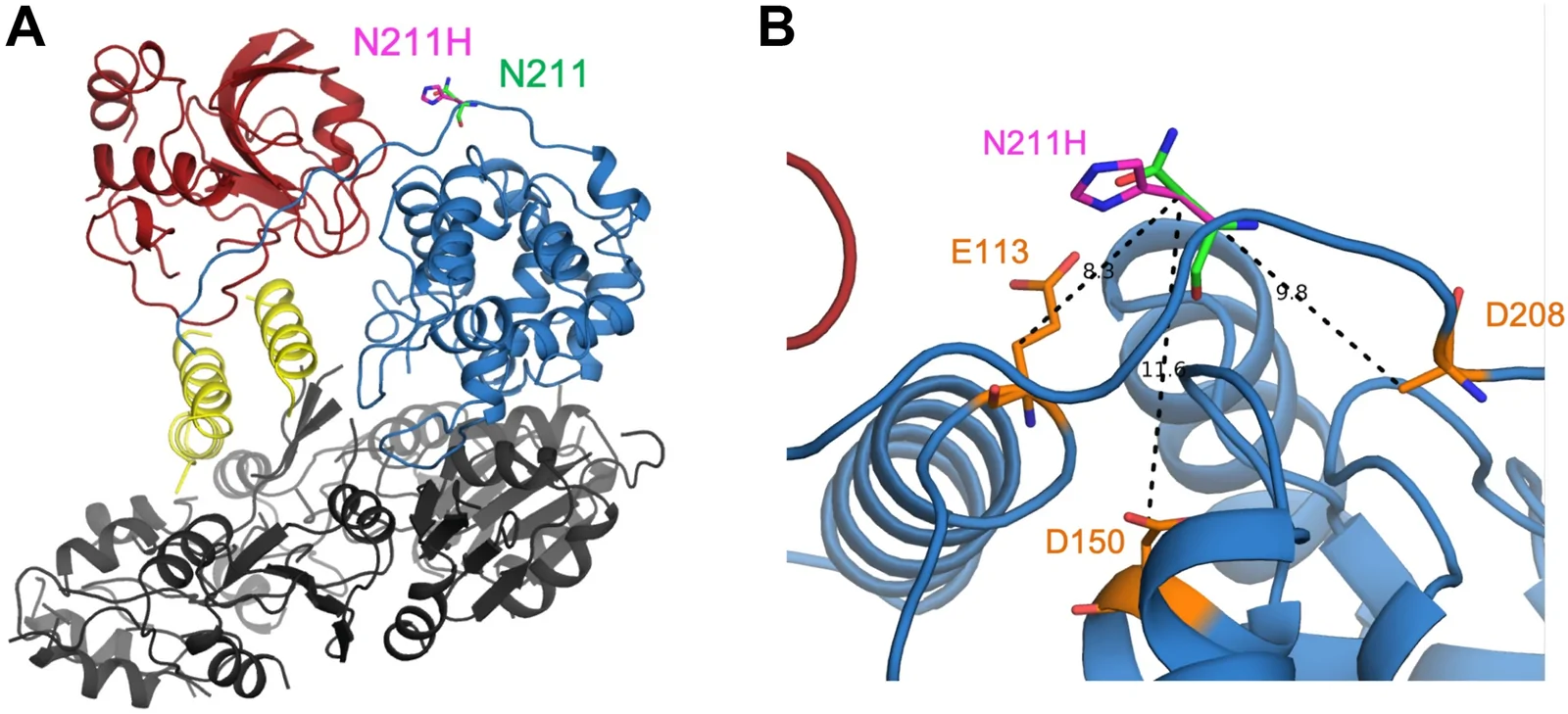
**Location of the PlyCA_N211H_ mutation and its potential interaction partners within the PlyC holoenzyme.***A,* the PlyC holoenzyme structure consists of eight PlyCB monomers that interact to form the CBD octamer (*gray*). The PlyCA subunit consists of an N-terminal GyH domain (*blue*), a central helical docking domain (*yellow*), and a C-terminal CHAP domain (*red*). The PlyCA_H211_ residue (*pink sticks*) was modeled into the PlyCA subunit and shown superimposed with the endogenous PlyCA_N211_ residue (*green sticks*). *B,* the β-carbon side-chain distance estimations between PlyCA_H211_ (*pink sticks*) and three nearby acidic amino acids that potentially interact electrostatically with PlyCA_H211_ are illustrated (*orange sticks*). CHAP, cysteine, histidine-dependent amidohydrolase/peptidase; GyH, glycosyl hydrolase; CBD, cell wall binding domain.

It remains elusive how the cationic properties of the PlyCA_H211_ side-chain stabilize the catalytic subunit of PlyC. One possibility is that PlyCA_H211_, which is located at the beginning of the flexible linker structure that interconnects the N-terminal GyH domain (Fig. 7*A*, blue) to the central helical docking domain (Fig. 7*A*, yellow), forms favorable electrostatic interactions with other localized anionic amino acids. To evaluate this model, the three acidic amino acids PlyCA_E113_, PlyCA_D150_, and PlyCA_D208_, all of which were within a predicted 12 Å radius of PlyCA_H211_, were identified as potential electrostatic interaction partners (Fig. 7*B*).

**Figure 8:**
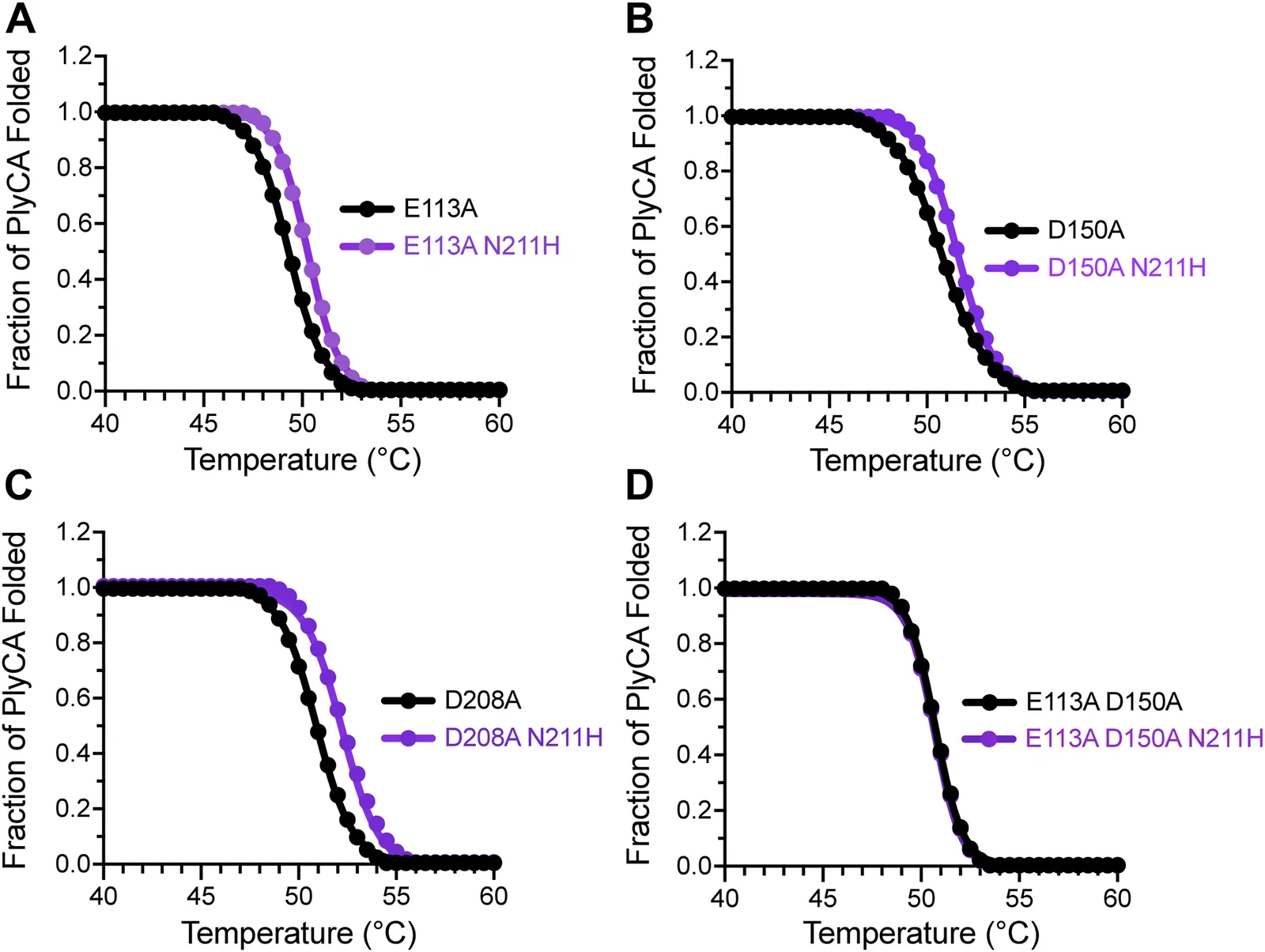
**Identifying the electrostatic interaction partners of PlyCA_H211_.** Using CD thermal denaturation experiments, the stabilizing potential of the PlyCA_N211H_ mutation was determined in the following backgrounds: *(A)* PlyC (PlyCA_E113A_), *(B)* PlyC (PlyCA_D150A_), *(C)* PlyC (PlyCA_D208A_), or *(D)* PlyC (PlyCA_E113A,D150A_). All experiments were performed in 20 mM sodium phosphate buffer, pH 7.0, using a heating rate of 1 °C/min. The structural transitions displayed correspond to the unfolding of the PlyCA subunit only. All protein samples analyzed were purified to homogeneity (Fig. S10). CD, circular dichroism.

To determine if PlyCA_E113_, PlyCA_D150_ and/or PlyCA_D208_ form stabilizing charge-mediated interactions with PlyCA_H211_, a two-part mutational thermal analysis was undertaken. First, the thermal stability of PlyC (PlyCA_E113A_), PlyC (PlyCA_D150A_), and PlyC (PlyCA_D208A_) was determined. Next, the PlyCA_N211H_ mutation was added to the aforementioned mutants in order to analyze whether this mutation had the same stabilizing potential as observed with the PlyC (PlyCA_N211H_) construct (PlyCA Δ*T*_*1/2*_ = +1.51 °C, Δ*T*_*G*_ = +1.99 °C at pH 7.0). Using 20 mM sodium phosphate buffer, pH 7.0, results from CD thermal denaturation experiments revealed adding the PlyCA_N211H_ mutation to PlyC (PlyCA_E113A_), PlyC (PlyCA_D150A_), and PlyC (PlyCA_D208A_) backgrounds increased the *T*_*1/2*_ of PlyCA by 0.92 °C (Fig. 8*A*), 0.80 °C (Figs. 8*B*) and 1.31 °C, respectively (Fig. 8*C*). A similar trend was observed when using DSC to analyze the thermal stability of the aforementioned mutants. Adding PlyCA_N211H_ to PlyC (PlyCA_E113A_), PlyC (PlyCA_D150A_), and PlyC (PlyCA_D208A_) backgrounds improved the respective *T*_*G*_ of PlyCA by 0.60 °C, 0.39 °C and 1.86 °C. As such, the PlyCA_D208A_ mutation had only a minimal impact on the stabilizing effect conferred by PlyCA_H211_, whereas the PlyCA_E113A_ and PlyCA_D150A_ mutations substantially reduced this stabilizing effect.

For the PlyC (PlyCA_N211H_) mutant, we hypothesized that the PlyCA subunit could be additively stabilized by the electrostatic fields formed between the cationic PlyCA_H211_ and the anionic PlyCA_E113_ and PlyCA_D150_ side chains. With this in mind, the stabilizing effect of the PlyCA_N211H_ mutation was evaluated in a PlyC (PlyCA_E113A,D150A_) background. CD thermal denaturation experiments analyzing PlyC (PlyCA_E113A,D150A_) and PlyC (PlyCA_E113A,D150A,N211H_) revealed near identical *T*_*1/2*_ values for PlyCA, with respective values of 50.72 °C and 50.69 °C (Fig. 8*D*). DSC experiments supported the CD findings, with PlyC (PlyCA_E113A,D150A_) and PlyC (PlyCA_E113A,D150A,N211H_) exhibiting *T*_*G*_ values for PlyCA of 47.52 °C and 47.59 °C, respectively (data not shown). These findings indicate that PlyCA_H211_ forms stabilizing electrostatic field interactions with both PlyCA_E113_ and PlyCA_D150_.

### Combining beneficial mutations from independent engineering approaches additively stabilizes PlyC

**Figure 9:**
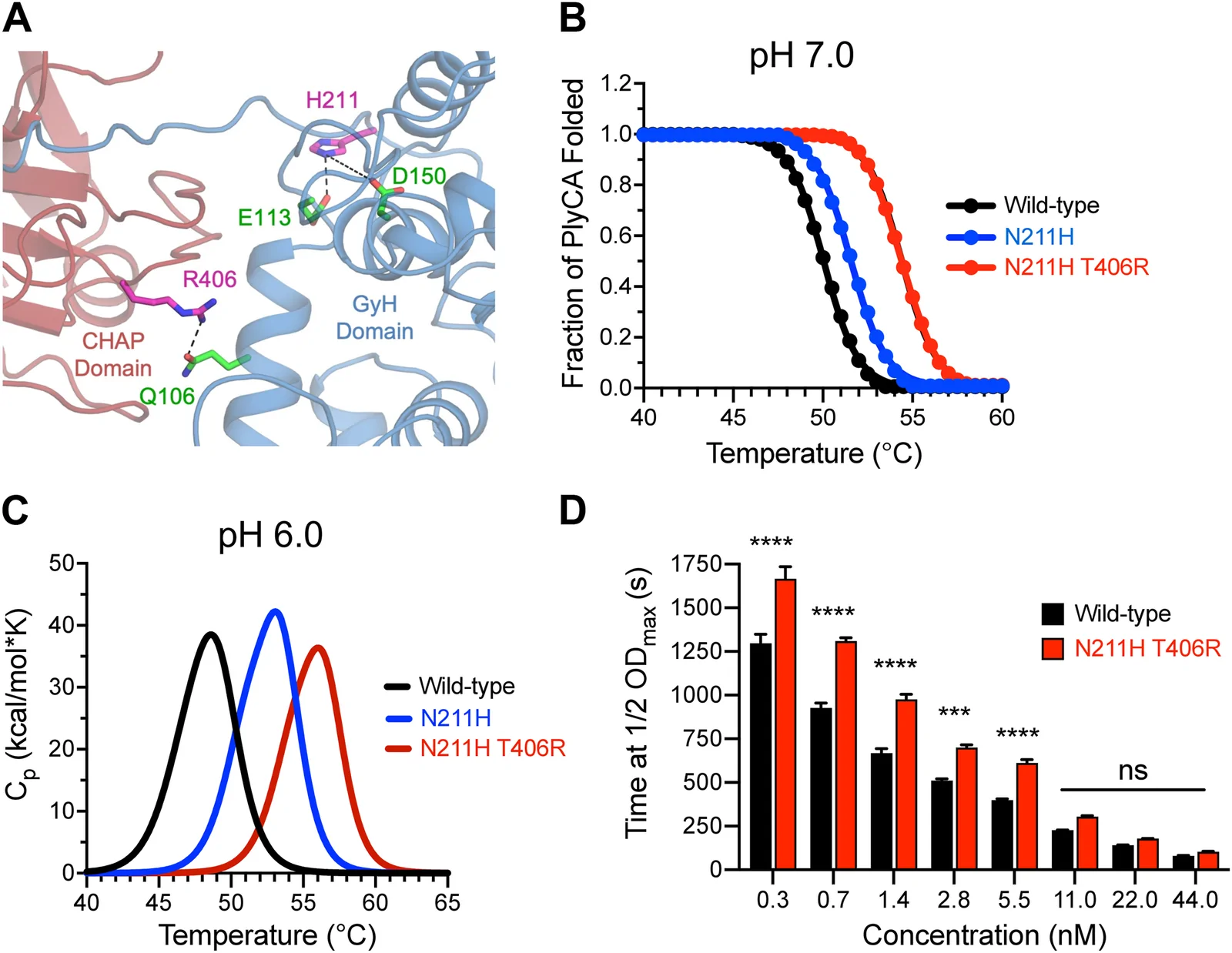
**Additive stabilizing effects when combining PlyCA_N211H_ and PlyCA_T406R_ mutations.***A,* the PlyCA_H211_ residue (*pink sticks, top*) was modeled into the linker structure stemming from the GyH domain (*blue*), with the proposed electrostatic interactions with PlyCA_E113_ and PlyCA_D150_ (*green sticks, top*) highlighted. The PlyCA_R406_ residue (*pink sticks, bottom*) was simultaneously modeled onto the surface of the CHAP domain (*red*), with the hypothesized interdomain hydrogen bond with PlyCA_Q106_ (*green sticks, bottom*) of the GyH domain shown. *B,* the structural stability of PlyC (PlyCA_N211H,T406R_) was measured in 20 mM sodium phosphate buffer, pH 7.0, using a heating rate of 1 °C/min, as measured by CD thermal denaturation experiments. The thermally induced structural transitions depicted correspond to the PlyCA subunit only. *C,* using DSC, the thermal stability of PlyC (PlyCA_N211H,T406R_) was evaluated in 20 mM sodium phosphate buffer, pH 6.0, using a heating rate of 1 °C/min. For each sample, the thermal transition corresponding to PlyCA unfolding is shown. *D,* using a dose-response turbidity reduction assay, the bacteriolytic activity of PlyC (PlyCA_N211H,T406R_) was compared to WT at equal molar concentrations. Activity was quantitated as the amount of time (s) required for each endolysin sample to decrease the initial optical density of the *S. pyogenes* culture by 50%. Error bars represent the ± standard error of the mean of triplicate experiments. *p-*values were calculated using a two-way ANOVA Sidak multiple comparison test. ns, not significant, ∗∗∗*p* < 0.0002, ∗∗∗∗*p* < 0.0001. All protein samples analyzed were purified to homogeneity (Fig. S11). For *B and C,* previously obtained data for WT PlyC and PlyC(PlyCA_N211H_) are shown for comparative purposes. CD, circular dichroism; CHAP, cysteine, histidine-dependent amidohydrolase/peptidase; DSC, differential scanning calorimetry; GyH, glycosyl hydrolase.

Previously, we reported findings from an *in silico* bioengineering study where the FoldX and Rosetta protein folding algorithms were used to improve the thermal stability of PlyC (26). In this study, each residue in the CHAP domain of PlyCA was individually mutated to the other 19 naturally occurring amino acids, with the resulting holoenzyme structures subsequently folded *in silico*. A list of potential stabilizing mutations was assembled based on the predicted folding free energy values, as well as visual inspection. One particular point mutation, PlyCA_T406R_, was experimentally validated to stabilize PlyCA by nearly 2.3 °C in 20 mM sodium phosphate buffer, pH 7.0, translating to a 16-fold increase in kinetic stability at 45 °C when compared to WT. PlyCA_H211_ and its hypothesized electrostatic interaction partners, PlyCA_E113_ and PlyCA_D150_, are located independently within the PlyCA structure when compared to PlyCA_R406_ and its predicted inter-domain hydrogen bonding partner, PlyCA_Q106_ (Fig. 9*A*).

The PlyCA_N211H_ and PlyCA_T406R_ mutations were combined to evaluate if the thermal stability of the endolysin could be additively enhanced. The thermal stability of PlyC (PlyCA_N211H,T406R_) was initially assessed in 20 mM sodium phosphate buffer, pH 7.0. Results from CD thermal denaturation experiments revealed the catalytic subunit of PlyC (PlyCA_N211H,T406R_) has a *T*_*1/2*_ of 54.25 °C, representing a 4.16 °C and 2.64 °C increase in structural stability compared to WT PlyC and PlyC (PlyCA_N211H_), respectively (Fig. 9*B*). Furthermore, DSC confirmed the thermal stability of the PlyCA subunit of PlyC (PlyCA_N211H,T406R_) was improved to 53.38 °C, corresponding to a 5.11 °C and 3.12 °C increase over WT PlyC and PlyC (PlyCA_N211H_), respectively (Table S2). Decreasing the buffer pH further enhanced the stabilizing effect of the two combined mutations. For example, at pH 6.0, the catalytic subunit of PlyC (PlyCA_N211H,T406R_) exhibited a *T*_*G*_ of 54.60 °C, a finding consistent with the enhanced protonation state of the PlyCA_N211H_ residue (Fig. 9*C*, Table S2). This thermal transition temperature represents a 7.46 °C and 3.36 °C improvement over WT (*T*_*G*_ = 47.14 °C) and PlyC (PlyCA_N211H_) (*T*_*G*_ = 51.24 °C) at pH 6.0, respectively.

For PlyC (PlyCA_N211H,T406R_), the effect of the two mutations on bacteriolytic activity was assessed using a dose-response turbidity reduction assay. Compared to WT PlyC, a modest but statistically significant reduction in activity was observed for PlyC(PlyCA_N211H,T406R_) at concentrations of 5.5 nM and below (Fig. 9*D*).

## Discussion

Antibiotic resistance is a current threat to public health and consequently presents a substantial clinical and economic burden to global health care systems (44). Phage endolysins represent alternative therapeutics currently under clinical development. However, for many of these enzymes, intrinsic thermolability remains a major limitation that can potentially impede downstream development. This characteristic is highly relevant to the practical development of protein therapeutics, as increased thermal stability can often enhance resistance to irreversible inactivation during manufacturing, storage, transport and formulation. Enhanced stability may also prolong the functional lifetime of the biologic under physiologically and environmentally relevant conditions. Accordingly, thermostabilization has been widely employed across diverse enzyme classes to improve developability and shelf life, even when catalytic activity itself remains unchanged.

In this study, we demonstrate a directed evolution methodology specific to increasing the thermal stability of bacteriolytic enzymes, such as endolysins (Fig. 2*A*). We further show that stabilizing mutations identified from two independent protein engineering approaches (*i.e.,* directed evolution and *in silico* computational screening) can be combined to have an additive effect on thermal stability (Fig. 9). These verified engineering methodologies, as well as the unique stabilizing mechanisms elucidated, can now be expanded to other thermolabile bacteriolytic enzymes for the purpose of increasing their intrinsic kinetic and thermal stability, ultimately improving their practical utility as antimicrobial biologics.

The streptococcal endolysin PlyC was used as a model for our directed evolution studies because the enzyme irreversibly unfolds and is kinetically inactivated at temperatures around 45 °C (27). The thermolability of PlyCA is believed to be a major contributor to limitations in long-term stability and formulation. We initially performed a comprehensive calorimetric analysis of all the structural components of the holoenzyme to strategically identify a thermolabile region of PlyC suitable for stabilization. Consistent with previous findings, data from these experiments confirmed that the PlyCB CBD is highly thermostable, whereas the PlyCA subunit is comparatively unstable (Table 1). Contrary to a previous hypothesis that implicated the C-terminal CHAP domain as the most thermolabile structural component of PlyC (27), results from our more comprehensive thermal analysis of PlyC suggest both the N-terminal GyH and C-terminal CHAP domains exhibit similar degrees of thermal instability.

It is worth noting that there are some additional discrepancies in the data obtained in this study compared to findings from a previous thermal analysis of PlyC (27). Examples include the thermal transition model used to fit the PlyC holoenzyme thermograms (five-state vs four-state), as well as the thermal stability of the isolated PlyCA (Δ*T*_*G*_ = 3.9 °C) and CHAP domain constructs (Δ*T*_*G*_ = 9.3 °C). The two unique thermal transition models used to fit the independent datasets may reflect differences in the sensitivity of the calorimeter and analysis software used. Additionally, variations in thermal transition temperatures can be explained by differences in buffer conditions. Our DSC studies were performed in 20 mM sodium phosphate buffer, pH 7.0, while the previous calorimetric analysis of PlyCA and the CHAP domain utilized PBS, pH 7.4. Disparities in buffer composition have been shown to affect the stability of PlyC structural components. For example, it was previously documented that the isolated PlyCB CBD exhibits thermal transitions at 70.4 °C and 83.5 °C in 20 mM sodium phosphate buffer, pH 7.4. The stability of the CBD was significantly increased in PBS, pH 7.4, with thermal transitions observed at 73.7 °C and 91.8 °C. Although still a hypothesis, the difference in thermal transition temperatures observed for PlyCA and the CHAP domain between the two studies can most likely be attributed to disparities between the pH and salt concentrations of the two buffer systems.

Directed evolution was applied to the entire catalytic subunit due to the global thermolability of PlyCA indicated by our calorimetric evaluation of the endolysin (Table 1). Using *plyCA* as a template for epPCR, a total of 18,000 mutants were screened over three rounds of directed evolution (Fig. 2). The lead candidate after the third round of screening, PlyC 33D2, consisted of the four PlyCA point mutations N211H, S224N, G249C, and A407S, each of which increased the kinetic stability of PlyC to varying degrees when isolated (Fig. 3, Table 2). Moreover, PlyCA_N211H_ was about 13-fold more kinetically-stabilizing than the other point mutations. PlyCA_N211H_ improved the *t*_*1/2*_ of the endolysin at 45 °C from 18 min (WT) to 335 min. Interestingly, the stabilizing potential of PlyCA_N211H_ was significantly reduced when combined with the other PlyC 33D2 point mutations; the mechanistic basis for this is still not understood. In addition to significantly increasing kinetic stability, the PlyCA_N211H_ point mutation enhanced the structural stability of the catalytic subunit by 1.52 °C in 20 mM sodium phosphate buffer, pH 7.0, while results from DSC showed a 1.99 °C improvement in thermal stability (Figs. 4, *A* and *B*, Table 3). The stabilizing effect of the point mutation was further amplified at a more acidic pH. For example, in 20 mM sodium phosphate buffer, pH 6.0, the structural stability of PlyC (PlyCA_N211H_) was improved 3.48 °C when compared to WT (Fig. 6*A*, Table 4), while DSC confirmed a 4.10 °C increase in thermal stability (Fig. 6*B*, Table 5). Although there is routinely a substantial reciprocity between enzymatic activity and thermal stability when thermostabilizing proteins (45, 46), PlyC (PlyCA_N211H_) did not exhibit any reduction in bacteriolytic activity when compared to WT (Fig. 4*C*). These collective findings resulted in PlyC (PlyCA_N211H_) being the lead candidate at the culmination of the directed evolution experiments.

Residue 211 of PlyCA is located at the beginning of the linker structure that interconnects the N-terminal GyH domain to the central helical docking domain (Fig. 7*A*). Based on side-chain distance estimations, three anionic amino acid candidates were identified (PlyCA_E113_, PlyCA_D150_, and PlyCA_D208_) that could feasibly interact electrostatically with the protonated PlyCA_H211_ side-chain (Fig. 7*B*). Although other charge-based interactions involving PlyCA_H211_ could contribute to stabilization, such as α-helix dipole effects, solvation, or van der Waals interactions, we hypothesized that an electrostatic attraction between PlyCA_H211_ and a nearby acidic residue represents the primary stabilizing force. Mutation of PlyCA_D208_ to PlyCA_D208A_ had a negligible effect on the stabilizing potential of PlyCA_H211_ (Fig. 8*C*). Nonetheless, PlyCA_D208_ and PlyCA_H211_ may still interact electrostatically to some degree; however, most significant stabilization effects are greatest when two interacting residues are distant in sequence. If PlyCA_D208_ and PlyCA_H211_ do interact through electrostatics, their close sequence proximity would have little effect on the global stability of the PlyCA subunit. In contrast, simultaneous mutation of two proximal acidic residues, PlyCA_E113A_ and PlyCA_D150A_, completely negated the stabilizing effect of PlyCA_H211_ (Fig. 8*D*). Moreover, the stabilizing potential of PlyCA_H211_ was also partially compromised in either a PlyC(PlyCA_E113A_) (Fig. 8*A*) or PlyC(PlyCA_D150A_) background (Fig. 8*B*). These findings, taken together, support the model that electrostatic fields formed between the cationic PlyCA_H211_ and the anionic PlyCA_E113_ and PlyCA_D150_ residues strengthen PlyCA stability in an additive manner, possibly due to anchoring a segment of the linker to the surface of the GyH domain. The enthalpic gains associated with this molecular reorganization may account for the observed improvement in stability.

Combining the PlyCA_N211H_ mutation identified through directed evolution with the stabilizing PlyCA_T406R_ mutation identified in a previous rational-based *in silico* screen of PlyC resulted in an additive enhancement of thermal stability. The catalytic subunit of PlyC(PlyCA_N211H,T406R_) was stabilized by 7.46 °C when compared to WT at pH 6.0 (Fig. 9*C*, Table S2). Although the double mutant exhibited enhanced thermal stability, a modest reduction in bacteriolytic activity was observed at lower enzyme concentrations, highlighting the balance between stability and catalytic performance that often accompanies protein engineering efforts (Fig. 9*D*).

For PlyC(PlyCA_N211H,T406R_), while the overall magnitude of a 7.46 °C increase in thermal stability may appear modest, such improvements are often considered meaningful in protein engineering because relatively small increases in unfolding temperature can substantially enhance resistance to irreversible inactivation. For PlyC, the catalytic PlyCA subunit unfolds at temperatures substantially below those required to destabilize the PlyCB octamer, making PlyCA the primary determinant of holoenzyme thermal tolerance. Consequently, even incremental stabilization of PlyCA represents progress toward improving the overall robustness and developability of the enzyme. Although additional studies are required to establish quantitative relationships between thermal stability, long-term stability and therapeutic performance, the engineering approaches described here provide a framework for addressing a key developability limitation of thermolabile endolysins.

Using the PlyC crystal structure as a guide, future rational-based engineering efforts can be implemented to further stabilize PlyCA. For example, the electrostatic fields formed between PlyCA_E113_-PlyCA_H211_ and PlyCA_D150_-PlyCA_H211_ could potentially be substituted with stronger, more enthalpically favorable covalent interactions. An inter-domain covalent bond could also be engineered to replace the hypothesized stabilizing hydrogen bond formed between PlyCA_Q106_ and PlyCA_R406_. Examples of alternative validated protein thermostabilization strategies that could be expanded to PlyC include (i) rigidifying PlyCA turn and loop structures through glycine to alanine or Xxx-to-proline mutations, (ii) inserting intra-domain disulfide bonds and salt-bridges, (iii) helix capping through the introduction of amino acids that interact with the α-helix dipole, (iv) replacing residues in left-handed helical structures with glycine or asparagine, (v) decreasing surface hydrophobicity, (vi) introducing charged residues to the surface to optimize the surface-charge network, and (vii) engineering clusters of aromatic-aromatic interactions (47, 48, 49, 50).

In conclusion, for the first time, we successfully validated the application of a directed evolution methodology to overcome the challenge of increasing the thermal stability of thermolabile bacteriolytic enzymes, such as endolysins. More explicitly, applying three rounds of directed evolution to the catalytic subunit of PlyC yielded a mutant with engineered electrostatic field interactions that significantly improved the kinetic and thermal stability of the enzyme. Combining favorable mutations independently identified from evolutionary and rational protein engineering methods additively enhanced the stability of PlyC to generate one of the more thermostable endolysins described from a mesophilic phage origin. The directed evolution and *in silico* screening methodologies discussed in this study can be further expanded to other thermolabile endolysins for the purpose of increasing long-term stability and downstream therapeutic applicability.

## Experimental procedures

### Bacterial strains and culture conditions

*S. pyogenes* D471 was routinely grown in Todd Hewitt broth with 1% (w/v) yeast extract as previously described (18, 51). *E. coli* strains were grown in Luria-Bertani (LB) broth at 37 °C with aeration unless otherwise stated. When needed, ampicillin (100 μg/ml) and/or chloramphenicol (35 μg/ml) were added to the media.

### Molecular cloning and site-directed mutagenesis

The *plyC* operon is composed of *plyCB*, a non-coding region termed the lysin intergenic locus, or *lil*, and *plyCA*. The *plyC* operon and *plyCA* alone were independently cloned into pBAD24, and *plyCB* was cloned into pBAD33 as previously described (22). For the other PlyC-related structural constructs, the standard 50 μl polymerase chain reaction (PCR) mixture consisted of 1 ng template DNA, 1x Q5 reaction buffer, 0.2 mM dNTP mix, 0.5 μM of each primer and 1 U of Q5 DNA polymerase (New England Biolabs). Using pBAD24::*plyC* as the template, *gyh* (GyH-F and GyH-R), *chap* (CHAP-F and CHAP-R), *plyCΔgyh* (PlyCB_lil-F, PlyCB_lil-R, PlyCΔGyH-F and CHAP-R) and *plyCΔchap* (PlyCB_lil-F and PlyCΔCHAP-R) were amplified using the primers listed in Table S3. *plyCΔgyh* was constructed by overlap-extension PCR with three sets of primers. Two PCR products were amplified with the PlyCB_lil-F/PlyCB_lil-R and PlyCΔGyH-F/CHAP-R primer pairs, respectively. The two PCR products were then fused together with PlyCB_lil-F and CHAP-R to generate full-length *plyCΔgyh*. Using *plyCΔgyh* as the template, *plyCΔΔ* was amplified with PlyCB_lil-F and PlyCΔΔ-R. Both *gyh* and the nucleotide sequence encoding the helical docking domain for the *plyCΔΔ* construct comprised a translated C-terminal 6x His-tag. The thermocycler heating conditions were 98 °C for 30 s, 35x (98 °C for 10 s, 60 °C for 30 s, 72 °C for 30 s/kb) and 72 °C for 2 min. The PCR products were double digested with MfeI and XbaI restriction endonucleases (New England Biolabs), ligated into pBAD24 using the EcoRI and XbaI restriction sites of the plasmid, and then transformed into *E. coli* DH5α. Following DNA sequencing, each plasmid construct was transformed into *E. coli* BL21(DE3)pLysS for protein expression.

### Protein expression and purification

The PlyC-related structural constructs and point mutants were expressed in LB supplemented with ampicillin (pBAD24) or chloramphenicol (pBAD33) at 37 °C with aeration (180 rpm). Once each culture was grown to mid-log phase, protein expression was induced with 0.25% (w/v) L-arabinose for 4 h. Cells were then harvested, washed and resuspended in PBS, pH 7.2, supplemented with 1 mM phenylmethanesulfonyl fluoride (Sigma-Aldrich). Bacteria were lysed *via* sonication. The resulting lysate was cleared by centrifugation at 13,000 RPM for 1 h at 4 °C and passed through a 0.2 μm syringe filter to generate the crude lysate. With the exception of GyH and PlyCΔΔ, each protein sample was purified to homogeneity as previously mentioned (18). GyH and PlyCΔΔ were purified using immobilized metal affinity chromatography. The crude lysates were applied to a Ni^2+^-charged Profinity immobilized metal affinity chromatography column (Bio-Rad) in PBS, pH 7.2, supplemented with 10 mM imidazole. The column was washed and protein samples were eluted using a 20 to 500 mM imidazole step gradient. Elution fractions containing highly pure GyH or PlyCΔΔ were dialyzed against PBS, pH 7.2, sterile-filtered, and stored at −80 °C.

When constructing the PlyC point mutants, mutations were introduced into *plyCA* using the Phusion Site-Directed Mutagenesis Kit (Thermo Fisher Scientific). Mutations were placed in the middle of the forward 30 nucleotide phosphorylated primer for each mutant, with the phosphorylated reverse primer being complementary to the next 30 nucleotides upstream. The standard 50 μl PCR reaction mixture consisted of 1 ng of pBAD24::*plyC*, 1x Phusion HF buffer, 0.2 mM dNTP, 0.5 μM of each primer, and 1 U of Phusion DNA polymerase. The thermocycler heating conditions consisted of 98 °C for 30 s, 25x (98 °C for 10 s, 65 °C for 30 s, 72 °C for 4 min) and 72 °C for 5 min. The mutagenized PCR products were ligated and transformed into *E. coli* DH5α. After confirming the mutations by DNA sequencing, each plasmid construct was transformed into *E. coli* BL21(DE3)pLysS for protein expression.

### Directed evolution methodology

epPCR using Mutazyme II (Agilent Technologies) was applied to *plyCA* at a low mutation rate of 2 to 3 nucleotides per *plyCA* gene. The standard 50 μl epPCR reaction mixture consisted of 3 μg of pBAD24::*plyCA* (750 ng of target DNA), 1x Mutazyme II reaction buffer, 0.2 mM dNTP mix, 0.5 μM of each primer (GyH-F and CHAP-R; Table S3), and 0.05 U of Mutazyme II DNA polymerase. The thermocycler heating conditions consisted of 95 °C for 2 min, 30x (95 °C for 30 s, 60 °C for 30 s, 72 °C for 2 min) and 72 °C for 10 min. The mutant *plyCA* library was then double digested with MfeI and XbaI restriction endonucleases and ligated into the EcoRI and XbaI restriction sites of pBAD24. The resulting pBAD24::*plyCA* mutant constructs were transformed into a genetically modified *E. coli* DH5α strain harboring pBAD33::*plyCB* and then plated on LB agar plates supplemented with ampicillin and chloramphenicol. Notably, the PlyC holoenzyme can be reconstituted when coexpressing PlyCA and PlyCB using the *E. coli* DH5α pBAD24::*plyCA*, pBAD33::*plyCB* double transformant (22).

The subsequent screening methodology was performed as previously described (25). To summarize, each well of a 96-well flat-bottom untreated polystyrene microtiter plate was filled with 200 μl LB supplemented with ampicillin and chloramphenicol. As controls, column 1 was inoculated with bacterial colonies encoding parental PlyC. Each remaining well was inoculated with an individual bacterial colony from the mutant library. The microtiter plates were incubated at 37 °C overnight with aeration (300 rpm). A replica plate was prepared by adding 180 μl LB with ampicillin and chloramphenicol to each well of a new 96-well microtiter plate. Next, 20 μl from each well of the overnight microtiter plate was transferred to the corresponding well of the replica plate. The original microtiter plate was stored at 4 °C until further needed. After a 1 h incubation at 37 °C with aeration, protein expression was induced by adding 0.25% (w/v) L-arabinose to each well of the replicate plate for 4 h. The microtiter plate was centrifuged for 10 min at 3000 rpm. The supernatant from each well was removed, and the bacteria were lysed by resuspension and incubation in 50 μl B-PER II Bacterial Protein Extraction Reagent (Thermo Fisher Scientific) for 20 min at room temperature. The total volume in each well was then increased to 120 μl using PBS, pH 7.2, and the insoluble lysate fraction was removed by centrifugation at 4 °C for 10 min at 3000 rpm. Next, 110 μl of the crude soluble lysate from each well was transferred to a 96-well thermocycler plate, except for the lysates located in wells A1 to D1. One hundred microliters of these four lysates containing overexpressed parental PlyC were transferred to the final 96-well flat-bottomed microtiter plate to serve as unheated positive controls for lytic activity. The 96-well thermocycler plate was incubated in a thermocycler at a previously optimized non-permissive temperature (see (25) for temperature optimization step; this temperature was 2–3 °C above the lowest temperature that inactivates the parental endolysin). Finally, 100 μl of the heat-treated soluble lysates was transferred to the final 96-well flat-bottomed microtiter plate. Residual bacteriolytic activity was measured against *S. pyogenes* using a turbidity reduction assay (see Measuring Endolysin Bacteriolytic Activity Against S. pyogenes below in Experimental procedures).

PlyC mutants preliminarily identified as displaying enhanced kinetic stability were re-screened using the same incubation temperature to eliminate false positives. Following the re-screening step, soluble lysates comprising overexpressed PlyC mutants with confirmed increased kinetic stability were then incubated over a wide range of temperatures using a gradient thermocycler. The PlyC mutant displaying residual bacteriolytic activity at the highest temperature was labeled as the lead candidate for that particular round of screening. The *E. coli* clone encoding the lead candidate was sequenced from the original microtiter plate in order to elucidate the mutation(s) conferring enhanced kinetic stability. Altogether, three rounds of directed evolution were performed, with a total of 18,000 PlyC mutants screened.

### Thermal kinetic inactivation experiments

An overnight culture of stationary-phase *S. pyogenes* was harvested, washed and resuspended in PBS, pH 7.2, to an initial OD_600_ of 2.0. Using an Echotherm benchtop heating block (Torrey Pines Scientific), WT PlyC and PlyC point mutants were incubated in triplicate at 5 μg/ml for a total of 3 h at 45 °C in PBS. At 20 min increments, an aliquot from each heated sample was removed and immediately incubated on ice for 5 min. Three adjacent wells of a 96-well flat-bottom untreated microtiter plate were filled with 100 μl of the cooled enzyme, followed by the addition of an equal volume of *S. pyogenes*. Using a SpectraMax M5 microplate reader (Molecular Devices), the residual bacteriolytic activity of the endolysin was analyzed by measuring the OD_600_ every 6 s for 20 min at 37 °C. Endolysin activity was equated to the V_max_ corresponding to the linear portion of the resulting decrease in optical density curve. Residual lytic activity was normalized to the activity displayed by the endolysin in the absence of heat treatment. All data were depicted as the ± standard error of the mean of triplicate experiments. The data points were fit using nonlinear or linear regression analysis (GraphPad Prism) in order to determine the *t*_*1/2*_ values.

### Measuring endolysin bacteriolytic activity against *S. pyogenes*

Turbidity reduction assays were used to quantitate the bacteriolytic activity of WT PlyC, PlyC(PlyCA_N211H_) and PlyC(PlyCA_N211H,T406R_) against *S. pyogenes*. In a 96-well flat-bottom untreated microtiter plate, purified endolysin at an initial concentration of 88 nM (10 μg/ml) was two-fold serially diluted in 100 μl of PBS, pH 7.2. An equal volume of *S. pyogenes* (see Thermal Kinetic Inactivation Experiments in *Materials and Methods* for cell preparation) was then added to each endolysin sample. Using a SpectraMax M5 microplate reader, the OD_600_ was immediately measured every 6 s for 30 min at 37 °C. The bacteriolytic activity of each sample was equated to the amount of time (s) required to reduce the initial OD_600_ by 50%. All data were depicted as the ± standard error of the mean of triplicate experiments. *p-*values were calculated using a two-way ANOVA Sidak multiple comparison test. ns, not significant, ∗∗∗*p* < 0.0002, ∗∗∗∗*p* < 0.0001.

### Circular dichroism

Far-UV and thermal denaturation CD experiments were performed using a Chirascan CD spectrometer (Applied Photophysics). To determine protein secondary structure composition, protein samples were analyzed at 0.1 mg/ml in 20 mM sodium phosphate buffer, pH 6.0 to 8.0. Using a 1 mm path length quartz cuvette, far-UV CD spectra from 190 to 260 nm were taken at 20 °C using 1 nm steps with 5 s signal averaging per data point. Spectra were collected in triplicate, followed by averaging, baseline subtraction, smoothing, and conversion to mean residue ellipticity by the Pro-Data software (https://photophysics.com/) (Applied Photophysics). Using a protein concentration of 1 mg/ml, thermal denaturation experiments consisted of monitoring the α-helical content of each sample at 222 nm while being heated from 20 °C to 95 °C at 1 °C/min in 20 mM sodium phosphate buffer, pH 6.0 to 8.0. The mean residue ellipticity was measured at 0.5 °C steps with 5 s signal averaging per data point. The data were smoothed, normalized, and fit with a Boltzmann sigmoidal curve. The first derivative of the melting curve was plotted to determine the *T*_*1/2*_ for each protein sample (52). For each CD thermal denaturation experiment, distinct thermally induced structural transitions were observed for both the PlyCA and PlyCB CBD components of the holoenzyme.

### Differential scanning calorimetry

Protein samples were calorimetrically analyzed using a Nano DSC (TA Instruments). After being degassed, all samples were maintained at a constant 3 atm of pressure over the duration of each experiment. The sample and reference cells consist of an optimal operational volume of 0.3 ml and were initially calibrated with equal volumes of sample buffer using three consecutive heating/cooling cycles at 1 °C/min over a temperature range of 15 °C to 105 °C. Using a molar concentration of 8.84 μM, protein samples were heated from 15 °C to 105 °C at 1 °C/min in 20 mM sodium phosphate buffer, pH 6.0 to 8.0, and immediately cooled over the same temperature range at 1 °C/min. Baseline subtraction, curve fitting, and calculation of *T*_*G*_ and *ΔH*_vH_ values were performed by the NanoAnalyze software (https://www.tainstruments.com/) (TA Instruments). In all DSC experiments that included the PlyCB CBD complexed to PlyCA (full-length, truncated, or point mutant(s)), thermal transitions were observed for both the PlyCA and PlyCB CBD components of the holoenzyme, with one exception. For the PlyCΔΔ construct, a distinct thermal transition for the catalytic subunit was not observed.

## Data availability

All data supporting the findings of this study are available within the article and its Supporting Information. Additional data are available from the corresponding authors upon reasonable request.

## Supporting information

This article contains supporting information.

## Conflict of interest

D. C. N. is a coinventor of patents related to the PlyC endolysin and a cofounder of ExoLytics, Inc., a company that develops endolysin-based technologies. No funding related to these interests supported the research reported in this manuscript.
