# Supplementary material for: Directed evolution of the bacteriophage endolysin PlyC reveals a charge-mediated mechanism of thermal stabilization
Source: J Biol Chem. 2026 Aug 5;302(9):113393. doi: 10.1016/j.jbc.2026.113393 (PMC13560338; doi:10.1016/j.jbc.2026.113393)
Supplement: Supplementary file 1 [file mmc1.docx]

**Supporting Information for**

Directed evolution of the bacteriophage endolysin PlyC reveals a charge-mediated mechanism of thermal stabilization

Ryan D. Heselpoth^1,2*^ and Daniel C. Nelson^1,3^

^1^ Institute for Bioscience and Biotechnology Research, University of Maryland, Rockville, MD 20850

^2^ Laboratory for Bacterial Pathogenesis and Immunology, The Rockefeller University, New York, NY 10065

^3^ Department of Veterinary Medicine, University of Maryland, College Park, MD 20742

*Correspondence: Ryan D. Heselpoth, [rheselpoth@rockefeller.edu](mailto:rheselpoth@rockefeller.edu); Daniel C. Nelson, [nelsond@umd.edu](mailto:nelsond@umd.edu)

**This file includes:**

Figures S1 to S11

Tables S1 to S3

Supplementary Figures

Figure S1. Electrophoretic analysis of the various purified PlyC structural constructs. The purity of each PlyC structural construct investigated in the thermal transition temperature study was analyzed using sodium dodecyl sulfate polyacrylamide gel electrophoresis (SDS-PAGE). The various lanes correlate with: (M) Molecular weight standard, (1) PlyC (PlyCA: 50 kDa, PlyCB: 8 kDa), (2) PlyCA (50 kDa), (3) PlyCB (8 kDa), (4) GyH domain (24 kDa), (5) CHAP domain (18 kDa), (6) PlyCΔGyH (PlyCA∆GyH: 26 kDa, PlyCB: 8 kDa), (7) PlyCΔCHAP (PlyCA∆CHAP: 33 kDa, PlyCB: 8 kDa), and (8) PlyCΔΔ (PlyCA∆∆: 7 kDa, PlyCB: 8 kDa).

Figure S2. DSC thermogram for PlyC. Using DSC, the thermal stability of PlyC at 8.84 μM was evaluated from 15°C to 105°C in 20 mM sodium phosphate, pH 7.0, using a heating rate of 1°C/min. Thermal transitions were fit using the NanoAnalyze software to obtain thermal transition temperatures. PlyC is best described by a five-state thermal transition model, with the first two thermal transitions corresponding to PlyCA unfolding and subsequent transitions corresponding to the PlyCB CBD.

Figure S3. DSC thermogram for PlyCA. Using DSC, the thermal stability of PlyCA at 8.84 μM was evaluated from 15°C to 105°C in 20 mM sodium phosphate, pH 7.0, using a heating rate of 1°C/min. Thermal transitions were fit using the NanoAnalyze software to obtain thermal transition temperatures. PlyCA is best described by a three-state thermal transition model.

Figure S4. DSC thermogram for the PlyCB CBD. Using DSC, the thermal stability of the PlyCB CBD at 8.84 μM was evaluated from 15°C to 105°C in 20 mM sodium phosphate, pH 7.0, using a heating rate of 1°C/min. Thermal transitions were fit using the NanoAnalyze software to obtain thermal transition temperatures. The PlyCB CBD is best described by a three-state thermal transition model.

Figure S5. DSC thermogram for the N-terminal GyH of PlyCA. Using DSC, the thermal stability of the N-terminal GyH domain of PlyCA at 8.84 μM was evaluated from 15°C to 105°C in 20 mM sodium phosphate, pH 7.0, using a heating rate of 1°C/min. Thermal transitions were fit using the NanoAnalyze software to obtain thermal transition temperatures. The GyH domain is best described by a three-state thermal transition model.

Figure S6. DSC thermogram for the C-terminal CHAP domain of PlyCA. Using DSC, the thermal stability of the C-terminal CHAP domain of PlyCA at 8.84 μM was evaluated from 15°C to 105°C in 20 mM sodium phosphate, pH 7.0, using a heating rate of 1°C/min. Thermal transitions were fit using the NanoAnalyze software to obtain thermal transition temperatures. The CHAP domain is best described by a three-state thermal transition model.

Figure S7. DSC thermogram for PlyCΔGyH. Using DSC, the thermal stability of PlyCΔGyH at 8.84 μM was evaluated from 15°C to 105°C in 20 mM sodium phosphate, pH 7.0, using a heating rate of 1°C/min. Thermal transitions were fit using the NanoAnalyze software to obtain thermal transition temperatures. PlyCΔGyH is best described by a five-state thermal transition model, with the first two thermal transitions specific to PlyCAΔGyH and the subsequent transitions corresponding to the PlyCB CBD.

Figure S8. DSC thermogram for PlyCΔCHAP. Using DSC, the thermal stability of PlyCΔCHAP at 8.84 μM was evaluated from 15°C to 105°C in 20 mM sodium phosphate, pH 7.0, using a heating rate of 1°C/min. Thermal transitions were fit using the NanoAnalyze software to obtain thermal transition temperatures. PlyCΔCHAP is best described by a five-state thermal transition model, with the first two thermal transitions specific to PlyCAΔCHAP and subsequent transitions corresponding to the PlyCB CBD.

Figure S9. DSC thermogram for PlyCΔΔ. Using DSC, the thermal stability of PlyCΔΔ at 8.84 μM was evaluated from 15°C to 105°C in 20 mM sodium phosphate, pH 7.0, using a heating rate of 1°C/min. Thermal transitions were fit using the NanoAnalyze software to obtain thermal transition temperatures. The DSC thermograms for PlyCΔΔ is best described by a simplified three-state thermal transition model, in which a distinct PlyCA unfolding transition could not be independently resolved.

Figure S10. Electrophoretic analysis of purified wild-type PlyC and PlyC point mutants. The purity of each enzyme was analyzed by SDS-PAGE. The lanes correlate to: (M) Molecular weight standard, (1) wild-type PlyC, (2) PlyC 33D2, (3) PlyC(PlyCA_N211H_), (4) PlyC(PlyCA_S224N_), (5) PlyC(PlyCA_G249C_), (6) PlyC(PlyCA_A407S_), (7) PlyC(PlyCA_N211A_), (8) PlyC(PlyCA_N211K_), (9) PlyC(PlyCA_N211D_), (10) PlyC(PlyCA_E113A_), (11) PlyC(PlyCA_E113A,N211H_), (12) PlyC(PlyCA_D150A_), (13) PlyC(PlyCA_D150A,N211H_), (14) PlyC(PlyCA_D208A_), (15) PlyC(PlyCA_D208A,N211H_), (16) PlyC(PlyCA_E113A,D150A_), and (17) PlyC(PlyCA_E113A,D150A,N211H_).

Figure S11. Electrophoretic analysis of purified wild-type PlyC, PlyC(PlyCA_N211H_) and PlyC(PlyCA_N211H,T406R_). The purity of each enzyme was analyzed by SDS-PAGE. The lanes correspond to: (M) Molecular weight standard, (1) wild-type PlyC, (2) PlyC(PlyCA_N211H_), and (3) PlyC(PlyCA_N211H,T406R_).

Supplementary Tables

Table S1. List of the different PlyC structural constructs thermally characterized by DSC.

| **Construct** | **Description** | **PlyCA**  **Residues** | **PlyCB Residues** |
| --- | --- | --- | --- |
| PlyC | Full-length PlyC holoenzyme | 1 to 465 (50 kDa) | 1 to 72 (8 kDa) |
| PlyCA | PlyCA catalytic domain only | 1 to 465 (50 kDa) | -------- |
| PlyCB CBD | PlyCB CBD octomer only | -------- | 1 to 72 (8 kDa) |
| GyH Domain | PlyCA GyH domain only | 1 to 205 (23 kDa) | -------- |
| CHAP Domain | PlyCA CHAP domain only | 300 to 465 (18 kDa) | -------- |
| PlyCΔGyH | PlyC with GyH domain deleted | 226 to 465 (26 kDa) | 1 to 72 (8 kDa) |
| PlyCΔCHAP | PlyC with CHAP domain deleted | 1 to 298 (33 kDa) | 1 to 72 (8 kDa) |
| PlyCΔΔ | PlyC with GyH and CHAP domains deleted | 226 to 288 (7 kDa) | 1 to 72 (8 kDa) |

Table S2. Calorimetric analysis of wild-type PlyC, PlyC(PlyCA_N211H_), and PlyC(PlyCA_N211H,T406R_) in 20 mM sodium phosphate, pH 6.0 and pH 7.0.

|  | **PlyC Construct** | ***T_G1_* (°C)** | ***T_G2_* (°C)** | **ΔH_vH1_ (kcal/mol)** | **ΔH_vH2_ (kcal/mol)** |
| --- | --- | --- | --- | --- | --- |
| pH 6.0 | Wild-type | 47.14 | 49.05 | 165.11 | 201.16 |
|  | N211H | 51.24 | 53.49 | 185.08 | 230.74 |
|  | N211H T406R | 54.60 | 56.57 | 187.71 | 242.82 |
| pH 7.0 | Wild-type | 48.27 | 50.67 | 171.23 | 205.09 |
|  | N211H | 50.26 | 52.46 | 199.25 | 240.14 |
|  | N211H T406R | 53.38 | 55.28 | 185.00 | 266.03 |

* Data displayed correlates to the PlyCA subunit only.

**Table S3.** Oligonucleotide primers used for PCR.

| **Primer Name** | **Nucleotide Sequence** |
| --- | --- |
| GyH-F | 5’-CTTCTTCAATTGATTATGAGTAAGAAGTATACACAACAACAATACG-3’ |
| GyH-R | 5’-CCCTCTAGATTAATGGTGATGATGATGATGAATTGAACCACCTG-3’ |
| CHAP-F | 5’-CTTCTTCAATTGATTATGTCACCAGGTCAAAACGATTTAGGGTCG-3’ |
| CHAP-R | 5’-CCCTCTAGATTATTTAAATGTTATCAAACCAGTTAGTG-3’ |
| PlyCB_lil-F | 5’-CTTCTTCAATTGATTATGAGCAAGATTAATGTAAACGTAG-3’ |
| PlyCB_lil-R | 5’-CTTCTCTCTGTTAGCTTCCATTAAATAAATTCTCCCTTTC-3’ |
| PlyCΔGyH-F | 5’-GAAAGGGAGAATTTATTTAATGGAAGCTAACAGAGAGAAG-3’ |
| PlyCΔCHAP-R | 5’-GGGTCTAGATTA AACGGTCGGTTTGTCTGGG-3’ |
| PlyCΔΔ-R | 5’-CCCTCTAGATTAGTGATGGTGATGGTGATGCGATCCCTGTAAGTTAA  CATCAGC-3’ |
